# Uptake, outcomes, and costs of implementing non-invasive prenatal testing for Down’s syndrome into NHS maternity care: prospective cohort study in eight diverse maternity units

**DOI:** 10.1136/bmj.i3426

**Published:** 2016-07-04

**Authors:** Lyn S Chitty, David Wright, Melissa Hill, Talitha I Verhoef, Rebecca Daley, Celine Lewis, Sarah Mason, Fiona McKay, Lucy Jenkins, Abigail Howarth, Louise Cameron, Alec McEwan, Jane Fisher, Mark Kroese, Stephen Morris

**Affiliations:** 1 Genetics and Genomic Medicine, UCL Institute of Child Health, London WC1N 3BH, UK; 2 Great Ormond Street Hospital for Children NHS Foundation Trust, London, UK; 3 Department of Statistics, Plymouth University, Plymouth, UK; 4 Department of Applied Health Research, University College London, London, UK; 5 North-East Thames Regional Genetics Laboratory, Great Ormond Street Hospital for Children NHS Foundation Trust, London, UK; 6 PHG Foundation, Cambridge, UK; 7 Department of Obstetrics and Gynaecology, Nottingham University Hospitals, Nottingham, UK; 8 Antenatal Results and Choices (ARC), London, UK

## Abstract

**Objective** To investigate the benefits and costs of implementing non-invasive prenatal testing (NIPT) for Down’s syndrome into the NHS maternity care pathway.

**Design** Prospective cohort study.

**Setting** Eight maternity units across the United Kingdom between 1 November 2013 and 28 February 2015.

**Participants** All pregnant women with a current Down’s syndrome risk on screening of at least 1/1000.

**Main outcome measures** Outcomes were uptake of NIPT, number of cases of Down’s syndrome detected, invasive tests performed, and miscarriages avoided. Pregnancy outcomes and costs associated with implementation of NIPT, compared with current screening, were determined using study data on NIPT uptake and invasive testing in combination with national datasets.

**Results** NIPT was prospectively offered to 3175 pregnant women. In 934 women with a Down’s syndrome risk greater than 1/150, 695 (74.4%) chose NIPT, 166 (17.8%) chose invasive testing, and 73 (7.8%) declined further testing. Of 2241 women with risks between 1/151 and 1/1000, 1799 (80.3%) chose NIPT. Of 71 pregnancies with a confirmed diagnosis of Down’s syndrome, 13/42 (31%) with the diagnosis after NIPT and 2/29 (7%) after direct invasive testing continued, resulting in 12 live births. In an annual screening population of 698 500, offering NIPT as a contingent test to women with a Down’s syndrome screening risk of at least 1/150 would increase detection by 195 (95% uncertainty interval −34 to 480) cases with 3368 (2279 to 4027) fewer invasive tests and 17 (7 to 30) fewer procedure related miscarriages, for a non-significant difference in total costs (£−46 000, £−1 802 000 to £2 661 000). The marginal cost of NIPT testing strategies versus current screening is very sensitive to NIPT costs; at a screening threshold of 1/150, NIPT would be cheaper than current screening if it cost less than £256. Lowering the risk threshold increases the number of Down’s syndrome cases detected and overall costs, while maintaining the reduction in invasive tests and procedure related miscarriages.

**Conclusions** Implementation of NIPT as a contingent test within a public sector Down’s syndrome screening programme can improve quality of care, choices for women, and overall performance within the current budget. As some women use NIPT for information only, the Down’s syndrome live birth rate may not change significantly. Future research should consider NIPT uptake and informed decision making outside of a research setting.

## Introduction

Non-invasive prenatal testing (NIPT), based on sequencing of cell-free DNA in maternal plasma, is a highly effective screening test for Down’s syndrome with sensitivities around 99% and false positive rates of less than 0.1%,^1^ in both high risk and general populations.^2^ ^3^ NIPT can also screen for trisomies 13 and 18 and sex chromosome aneuploidies, albeit with poorer performance.^1^ In the United States, widespread uptake of NIPT has significantly reduced rates of invasive testing.^4^ NIPT is now available worldwide, but largely through private sector healthcare providers or partial self funding by women in public sector Down’s syndrome screening pathways in some European countries.^5^ Women and health professionals welcome the possibility of NIPT because of the potential for increased detection of Down’s syndrome pregnancies with a decreased need for invasive diagnostic testing and the associated iatrogenic miscarriages.^6^ ^7^ ^8^ However, implementation into public sector maternity care requires careful evaluation because of the likely changes to the care pathway, educational requirements, and potential effect on limited public sector resources. Several studies have suggested that NIPT for Down’s syndrome as a contingent test is likely to be cost effective.^9^ ^10^ ^11^ ^12^ ^13^ ^14^ ^15^ ^16^ ^17^ ^18^ ^19^ ^20^ ^21^ ^22^ The available UK data describing uptake of NIPT come from two maternity units where the uptake of Down’s syndrome screening is much higher (98.2%) than the national average of 66% for both the first trimester combined test and the second trimester quadruple test.^23^ ^24^ ^25^ Little information from the United Kingdom or elsewhere describes uptake of NIPT in units with a wider range of screening uptakes that would be more compatible with the national average, and none describes costs as well as outcomes in a fully state funded healthcare setting. Many national and international bodies recommend the use of NIPT as a screening test for women at increased risk of Down’s syndrome^26^ ^27^ ^28^; although some suggest it should be available to all women,^29^ ^30^ the current high cost of NIPT is likely to preclude this in most public sector screening programmes.^9^ ^31^

The objective of this study was to investigate the potential costs and consequences of introducing NIPT in our public sector Down’s syndrome screening pathway as a further screening test contingent on the risk generated by current screening, to help to inform the UK National Screening Committee’s decisions on implementation. We developed health economic models that were informed by national datasets and data from our cohort study, which provided information on uptake of Down’s syndrome screening, NIPT, and invasive testing, as well as pregnancy outcomes and costs. We chose to introduce NIPT as a contingent test for women with a current risk on screening of at least 1/1000 to allow evaluation of a range of potentially affordable policies and to avoid any possible reduction in detection of Down’s syndrome as suggested by previous studies.^9^ The current cost of NIPT precluded further lowering of the risk threshold in this study or offering NIPT to all women.

## Methods

### Study design and population

This RAPID (Reliable, Accurate Prenatal, non-Invasive Diagnosis) research programme study was a prospective cohort study performed in eight NHS hospitals between 1 November 2013 and 28 February 2015. However, it includes only cases reported over a 15 month period as the study was suspended from 26 September 2014 to 10 October 2014 owing to failure and subsequent unavailability of Illumina library preparation kits. NIPT was not available through NHS pathways in the maternity units participating in the study, but it was available through the private sector across the United Kingdom at the time the study was performed. In line with current clinical practice, all pregnant women attending for antenatal care were offered screening for Down’s syndrome by the combined test if they booked before 14 weeks’ gestation or the quadruple test if they booked later, up to 20 weeks. Women aged over 16 years accepting combined or quadruple testing with a subsequent risk of at least 1/1000 were eligible for the study. Women with a risk above 1/150, the current risk threshold for offering invasive diagnostic testing, were prospectively offered invasive testing or NIPT as a contingent test and could choose one of these options or no further testing (fig 1). If the risk was between 1/151 and 1/1000, they were offered NIPT. Women declining screening and those with a risk below 1/1000, with multiple pregnancies, or who could not understand the participant information were excluded. Figure 1 shows the study pathway, and a detailed protocol has been published.^32^ Participating maternity units had populations of pregnant women from a range of social and ethnic groups, with variable uptake of screening, and delivery of the screening pathway through either one stop or two stop clinics (see table 1 for details). Before starting the study, researchers trained local midwives by using materials developed on the basis of previous work.^8^ ^33^

**Figure f1:**
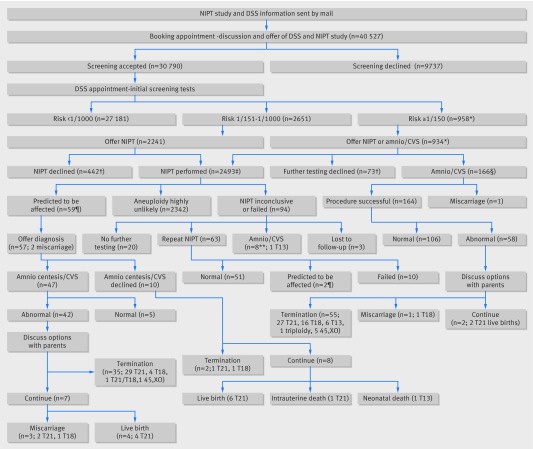
**Fig 1** Flowchart showing numbers of women recruited and outcomes. CVS=chorionic villus sampling; DSS=Down’s syndrome screening; NIPT=non-invasive prenatal testing; T13=trisomy 13; T18=trisomy 18; T21=trisomy 21. *Including 15 women with risk ≥1/150 for T13/T18 and risk 1/151-1/1000 for T21. †Some women underwent DSS and declined further testing but were known to have had NIPT in private sector (n=37). ‡One additional woman accepted NIPT but no blood sample was obtained. §One procedure was not possible. ¶Two women initially had inconclusive NIPT results but were positive on repeat testing; these are included in inconclusive/failed pathway and also predicted to be affected pathway. **Includes one case (T13) that had amniocentesis later in pregnancy after detection of fetal abnormalities

**Table 1 tbl1:** Details of women booked, uptake of Down’s syndrome screening, non-invasive prenatal testing, and invasive prenatal diagnosis before and during study for all eight participating maternity units. Values are numbers (percentages)

	Maternity units*								Total
	UCLH†	QHR	SGH*	SAL	PAHS	TAY	QCCH	WHIT	
**November 2011 to October 2012**									
Total booked	6456	11 669	6179	3057	6541	3666	5300	5066	47 934
DS screening uptake	5647 (87.5)	9712 (83.2)	5670 (91.8)	1390 (45.5)	4424 (67.6)	2759 (75.3)	4555 (85.9)	3575 (70.6)	37 732 (78.7)
Risk ≥1/150	216 (3.8)	242 (2.5)	164 (2.9)	29 (2.1)	128 (2.9)	98 (3.6)	121 (2.7)	113 (3.2)	1111 (2.9)
IPD uptake risk ≥1/150	153 (71)	99 (41)	116 (71)	17 (59)	80 (63)	50 (51)	78 (64)	74 (65)	667 (60.0)
Risk ≥1/150 declined all testing	63 (29)	143 (59)	48 (29)	12 (41)	48 (38)	48 (49)	43 (36)	39 (35)	444 (40.0)
**Study period (1 November 2013 to 28 February 2015)**									
Total booked	10 130	10 441	6011	2732	4117	1946	2988	2162	40 527
DS screening uptake	7497 (74.0)	8829 (84.6)	5436 (90.4)	1196 (43.8)	3015 (73.2)	1099 (56.5)	2138 (71.6)	1580 (73.1)	30 790 (76.0)
Total risk >1/1000	1199 (16.0)	735 (8.3)	611 (11.2)	129 (10.8)	354 (11.7)	76 (6.9)	297 (13.9)	208 (13.2)	3609 (11.7)
Total risk ≥1/150§	289 (3.9)	216 (2.4)	142 (2.6)	31 (2.6)	92 (3.1)	36 (3.3)	96 (4.5)	56 (3.5)	958 (3.1)
Total risk 1/151-1/1000	910 (12.1)	519 (5.9)	469 (8.6)	98 (8.2)	262 (8.7)	40 (3.6)	201 (9.4)	152 (9.6)	2651 (8.6)
Eligible and offered risk >1/1000	1161 (15.5)	606 (6.9)	565 (10.4)	93 (7.8)	287 (9.5)	68 (6.2)	264 (12.3)	131 (8.3)	3175 (10.3)
Eligible and offered risk ≥1/150§	281 (3.7)	212 (2.4)	138 (2.5)	29 (2.4)	90 (3.0)	36 (3.3)	92 (4.3)	56 (3.5)	934 (3.0)
Eligible and offered risk >1/151-1/1000	880 (11.7)	394 (4.5)	427 (7.9)	64 (5.4)	197 (6.5)	32 (2.9)	172 (8.0)	75 (4.7)	2241 (7.3)
IPD uptake risk ≥1/150	53 (19)	29 (14)	30 (22)	1 (3)	16 (18)	7 (19)	14 (15)	16 (29)	166 (17.8)
NIPT uptake risk ≥1/150	211 (75)	157 (74)	96 (70)	28 (97)	67 (74)	27 (75)	72 (78)	37 (66)	695 (74.4)
NIPT uptake risk 1/151-1/1000	803 (91.3)	280 (71)	360 (84)	49 (77)	107 (54)	20 (63)	129 (75)	51 (68)	1799 (80.3)
Declined all testing risk ≥1/150¶	17 (6)	26 (12)	12 (9)	0 (0)	7 (8)	2 (6)	6 (7)	3 (5)	73 (7.8)
Declined all testing risk 1/151-1/1000¶	77 (8.8)	114 (29)	67 (16)	15 (23)	90 (46)	12 (38)	43 (25)	24 (32)	442 (19.7)

DS=Down’s syndrome; IPD=invasive prenatal diagnosis; NIPT=non-invasive prenatal testing.

*UCLH=University College London Hospitals NHS Foundation Trust (started 1 Nov 2013); QHR=Barking, Havering and Redbridge University Hospitals NHS Trust (started 20 Jan 2014); SGH=St George's University Hospitals NHS Foundation Trust (started 3 Feb 2014); SAL=Salisbury NHS Foundation Trust (started 27 Jan 2014); PAHS=University Hospital Southampton NHS Foundation Trust (started 2 Jun 2014); TAY=NHS Tayside (started 4 Aug 2014); QCCH=Imperial College Healthcare NHS Trust (started 4 Aug 2014); WHIT=Whittington Hospital NHS Trust (started 11 Aug 2014).

†One stop clinic (combined test results on day of scan so that NIPT is usually discussed at same visit as receiving DS screening result).

‡Staged start to recruitment and not all sites were recruiting until 11 Aug 2014, and study was suspended from 26 Sept 2014 to 10 Oct 2014 owing to failure and subsequent unavailability of library preparation kits. Thus, data presented here cover 15 month period.

§Including 15 women with risk ≥1/150 for trisomy 18/13 and risk 1/151-1/1000 for trisomy 21.

¶13 women with risk ≥1/150 and 24 women with risk 1/151-1000 underwent DS screening and declined NIPT (and IPD if high risk) but were known to have had NIPT in private sector (n=37).

During the study, all pregnant women were sent information on NIPT with their booking information (appendix A). While booking women for maternity care, midwives briefly discussed NIPT and obtained permission to contact the woman if her Down’s syndrome screening result was a risk of at least 1/1000. Women with a risk of 1/1000 or greater were given more detailed participant information (appendix B) and offered an appointment with a healthcare professional trained to discuss the benefits and limitations of NIPT before giving written consent to participate in the study. At sites with one stop Down’s syndrome screening, this offer of NIPT was usually made on the same day that screening was performed. At other sites, women were contacted by phone and offered an appointment to discuss NIPT. For those who could not be contacted, two further attempts were made before they were excluded from the study. Women with a risk of at least 1/150 and a nuchal translucency measurement of at least 3.5 mm, or sonographic abnormalities, were offered a choice between NIPT (including testing for Turner’s syndrome), invasive prenatal diagnosis, or neither. All women consenting to NIPT had blood taken for sequencing of cell-free DNA in our public sector accredited genetics laboratory, using a HiSeq 2500 (Illumina Inc) and locally developed protocols and bioinformatics pipeline.^34^ Analysis of chromosomes 21, 18, and 13 was performed in all cases; the X chromosome was included in the analysis only for women at high risk with a nuchal translucency measurement of at least 3.5 mm or sonographic abnormalities. NIPT results were reported as “highly unlikely to be affected” or “predicted to be affected”. We aimed to report NIPT results within 10 days of blood draw. All women with inconclusive or failed tests were offered repeat NIPT. Those with a Down’s syndrome screening risk of at least 1/150 were offered the choice of repeat NIPT or invasive testing. Women with a “predicted to be affected” result were advised that although the NIPT result was positive, it is not diagnostic and invasive testing for confirmation is recommended. Pregnancy outcomes were ascertained from local unit records or the women themselves. To determine any change in women’s choices, we compared uptake of Down’s syndrome screening and uptake of direct invasive testing in women with a risk of at least 1/150 for the period 1 November 2011 to 31 October 2012, before the availability of NIPT in the United Kingdom, and during the study.

### Sample size calculation

The overall study sample size was justified on the precision achieved for the differences in uptake of testing, overall detection rate, and false positive rate between the existing Down’s syndrome screening pathway and the new contingent NIPT pathway. Initial estimates indicated that a screened sample of 25 000 would enable a reduction in false positive rate with the contingent pathway compared with the existing pathway to be estimated to within ±0.2%.^32^

### Measuring costs and consequences

We did a cost consequence analysis of NIPT as contingent testing from the perspective of the UK National Screening Committee, as this committee will advise government ministers whether to implement NIPT into NHS care.^35^ We chose this form of economic evaluation because there are several relevant outcome measures (number of invasive tests, Down’s syndrome cases detected, invasive testing related miscarriages). Quality adjusted life years (QALYs) are not commonly used in economic evaluations of prenatal testing for Down’s syndrome, and we did not use them here. We therefore limited the analysis to the costs of the screening pathway (screening, NIPT, and diagnostic testing, including sampling, laboratory testing, and feeding back the results). Costs were expressed in 2012/13 UK£; the time horizon was the duration of pregnancy, so discounting was unnecessary. We developed a decision tree (fig 2) for four different screening pathways (current pathway and NIPT as a contingent screening test for women with a screening risk of ≥1/150, ≥1/500, and ≥1/1000) and populated it with data from the cohort study and other sources. In our study, women with a risk of at least 1/150 could choose to go directly to invasive testing, and we included three additional scenarios in which invasive testing was allowed only after a positive NIPT result. This yields a total of seven testing strategies being compared. Although NIPT may have a role in detecting other trisomies, the overarching aim of the RAPID evaluation study was to evaluate NIPT for Down’s syndrome. For the economic analysis, we therefore focused on the Down’s syndrome screening pathway only and did not include other trisomies, even though women were offered screening for trisomies 13 and 18 in some clinics. Appendix C gives further details about the cost consequences analysis.

**Figure f2:**
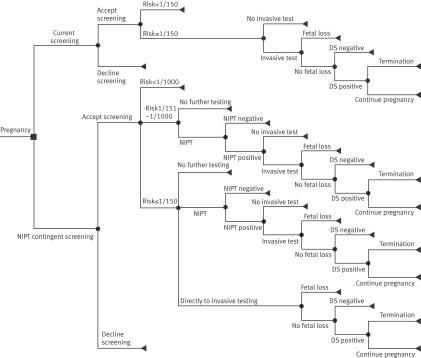
**Fig 2** Decision tree depicting current screening pathway and new pathway using non-invasive prenatal testing (NIPT) as contingent screening. In current screening pathway, women are offered invasive testing when their risk based on combined or quadruple test is ≥1/150. Small risk of procedure related miscarriage exists, so some women at high risk decide not to undergo any further testing. If result of invasive test is positive, women can decide to terminate pregnancy. In NIPT pathway, women are offered NIPT test after high risk result (depending on threshold: ≥1/150, ≥1/500, or ≥1/1000) based on combined or quadruple test. Pathways for different thresholds are similar except for threshold risk at which NIPT is offered as contingent screening. If NIPT test result is positive, invasive test is offered to confirm diagnosis. Some women with risk ≥1/150 after combined or quadruple test might decide to have invasive test directly and not have NIPT first. DS=Down’s syndrome

#### Model inputs

We populated the decision tree by using two datasets (table 2). The first was based predominantly on data from the RAPID study. The second was based predominantly on national data, using data from the RAPID study mainly to quantify test uptake behaviours associated with NIPT.

**Table 2 tbl2:** Input parameters for analysis of benefits and costs of non-invasive prenatal testing for aneuploidy as contingent test

Parameter	RAPID data	National data
**Screening test performed (%)**		
Combined test (first trimester)	88.5*	86.9^25^
Quadruple test (second trimester)	11.5*	13.1^25^
**Uptake (%)**		
DSS—current pathway	78.7*	66.2^25^
DSS—NIPT pathway	78.7†	66.2†
NIPT after ≥1/150 risk (NIPT pathway)	72.5‡	72.5‡
NIPT after 1/151-1/1000 risk (NIPT pathway)	70.5‡	70.5‡
NIPT after ≥1/150 risk—no direct IPD	91.0‡	91.0‡
IPD after positive screening (current pathway)	54.0‡	54.0‡
Directly to IPD after ≥1/150 risk (NIPT pathway)	20.0‡	20.0‡
IPD after positive NIPT	80.4*	80.4*
**Test outcomes (%)**		
Women with DSS risk ≥1/150	2.7*	2.3§
Women with DSS risk 1/151-1/500	3.2*	3.4§
Women with DSS risk 1/151-1/1000	7.1*	7.5§
NIPT positive if both DSS ≥1/150 and accepted NIPT	4.0‡	4.0‡
NIPT positive if both DSS 1/151-1/500 and accepted NIPT	0.38¶	0.38¶
NIPT positive if both DSS 1/151-1/1000 and accepted NIPT	0.25¶	0.25¶
NIPT positive if both DSS ≥1/150 and accepted NIPT—no direct IPD	7.9‡	7.9‡
NIPT repeat test	1.2*	1.2*
IPD positive if accepted IPD (current pathway)	10.1‡	10.1‡
IPD positive if accepted IPD after positive NIPT	90.1*	90.1*
IPD positive—directly to IPD ≥1/150 (NIPT pathway)	21.9‡	21.9‡
IPD related miscarriage	0.5^36^	0.5^36^
**Costs (£)**		
Combined test	27.52^37^	27.52^37^
Quadruple test	37.20^37^	37.20^37^
NIPT		
Laboratory costs NIPT**	250*	250*
Costs midwife for counselling and feedback	15.96^38^	15.96^38^
Costs phlebotomy and sending in sample	9^39^	9^39^
Cost of invasive test††	650*	650*

Two separate analyses were undertaken, one using RAPID data and second using national data to estimate performance for whole country. Where RAPID data were not available, national data were used and vice versa.

DSS=Down’s syndrome screening; IPD=invasive prenatal diagnosis; NIPT=non-invasive prenatal testing.

*Data taken directly from RAPID study.

†Uptake of screening in RAPID clinics was slightly lower during RAPID study than before NIPT was offered (76.0% *v* 78.7%). Analysis assumed that screening uptake would not change because of NIPT.

‡Modelled figures based on RAPID data, national prevalence, and maternal age distribution of England and Wales.

§Age standardised rates for 2011 reference population.

¶National data, because numbers in RAPID study were too low to give reliable results.

**Includes labour, DNA extraction, quality control, library preparation, HiSeq reagents, plasticware, symphony service contract, HiSeq service contract, Qiacube service contract, 20% consumables VAT, 15% repeat/failure rate, and freezer storage costs.

††Weighted mean unit cost of chorionic villus sampling/quantitative fluorescence-polymerase chain reaction and amniocentesis/full karyotype reported by eight participating units, including pre-test counselling, consultant obstetrician time to perform procedure, cytogenetic laboratory costs, and post-test feedback and counselling.

*RAPID data*—We used data from the study to assess the uptake of screening, uptake of NIPT and invasive testing, and test outcomes. We assessed the uptake of screening, screening test results, and uptake of invasive testing in the current pathway by using a historical dataset collected retrospectively in 2011-12 in the same clinics that were involved in the RAPID study but before NIPT was available in the United Kingdom. Uptake of screening was slightly higher before NIPT was offered than during the RAPID study (78.7% *v* 76.0%), possibly because some women had NIPT in the private sector and subsequently opted out of the national programme. In this analysis, we therefore assumed that this decrease in uptake of screening was unrelated to the RAPID study, so we used the same uptake percentage as in the current programme (78.7%). As the RAPID data are based on relatively small numbers, data on uptake of NIPT, invasive testing, and the remaining test outcomes are based on RAPID data, but these were adjusted to account for the national prevalence of Down’s syndrome and maternal age distributions in England and Wales. This enabled valid comparison of different contingent screening strategies against a common underlying population (see appendices C and D for further details). For uptake of NIPT with a risk of Down’s syndrome of at least 1/150, the unadjusted figure from the RAPID study was 74.4% and the adjusted figure was 72.5%. For uptake of NIPT with a risk of 1/151 to 1/1000, the unadjusted figure from the RAPID study was 80.3% and the adjusted figure was 70.5%. We used published estimates for the rate of miscarriage related to invasive testing.^36^

*National data*—We ran another version of the analysis using predominantly national data to estimate costs on a national basis. We used national data to assess uptake of screening, screening test uptake in the current pathway, screening test outcomes, and the invasive testing related miscarriage percentage. Data on uptake of NIPT and direct invasive testing and the remaining test outcomes were based on RAPID data, adjusted as described above, as these data are not available from national sources.

In our study, there were five discordant positive NIPT results, giving a positive predictive value of NIPT of 90.9% for pregnancies with confirmed outcome, which is compatible with published data.^1^ ^2^ We therefore used this value and varied the positive predictive value of NIPT in the sensitivity analysis. For the additional scenarios, in which we did not allow the option of direct invasive testing for women with a risk of at least 1/150, we assumed that these women would have NIPT, which would give an overall uptake of NIPT in this group of 91%. In the RAPID study, several women at very high risk underwent invasive testing directly, so the proportion of positive NIPT results was expected to be higher when these women were to undergo NIPT first. We estimated an average of 7.9% positive NIPT results for this scenario.

We calculated the costs of Down’s syndrome screening, NIPT, and invasive testing on the basis of published sources and costs in participating centres (table 2).^25^ ^36^ ^37^ ^38^ ^39^

#### Model outputs

Outcomes were the number of invasive tests performed, the number of Down’s syndrome positive cases detected (by NIPT or direct invasive testing and confirmed by invasive testing or at birth), and the number of invasive testing related miscarriages. Costs are presented separately and combined for Down’s syndrome screening, NIPT, and invasive testing. Using the results of the study, we calculated estimates of the outcomes for NIPT in the Down’s syndrome screening programme, for a screening population of 698 500 annual live births in England and Wales.^40^ Detection of other trisomies is reported, but the economic analysis focuses on detection of Down’s syndrome to reflect the UK antenatal screening programme at the outset of the study.

#### Sensitivity analysis

We investigated uncertainty around model inputs by using one way sensitivity analysis and probabilistic sensitivity analysis (appendix C). In the one way sensitivity analysis, we varied one parameter at a time over a plausible range to identify the maximum value at which introducing NIPT to the national screening programme would be cost neutral. We calculated costs in high risk pregnancies (≥1/150) for a range of uptake values for NIPT and direct invasive testing. We also calculated the incremental costs of implementing NIPT at a range of laboratory cost values (£50 to £500). In the probabilistic sensitivity analysis, we produced 1000 simulations of the outputs, based on drawing random samples from the probability distributions of all input parameters. We used this to calculate 95% credible intervals for each model output. We also calculated costs and consequences separately for one stop (where NIPT occurs on the same day as Down’s syndrome screening) and two stop clinics.

### Patient involvement

Patients and the public are represented in the RAPID programme through the involvement of Genetic Alliance UK and the patient support charity Antenatal Results and Choices (ARC), which supports women undergoing prenatal testing. The director of ARC was a co-applicant on the funding application for the study reported here and involved in the study design, conduct, analysis, and reporting. During the preparation for the study, patients and their partners were consulted on the format and content of the information for patients to be used in the study. This was revised in the light of feedback from patients. The ARC director was involved in developing the education packages for health professionals and assisted with training to ensure that health professionals understood the needs of expectant parents. Preliminary results of the study have already been presented at relevant annual meetings of lay groups. More details will be included on the RAPID website once the study is published. We have acknowledged the contribution of parents to the study. The study was designed to identify parents’ priorities, experience, and preferences in order to inform appropriate implementation into the clinical care pathway.

## Results

### Sample characteristics and testing choices

During the study period, 40 527 pregnant women booked for maternity care at units participating in the study and 30 790 (76.0%) opted for Down’s syndrome screening. Overall, of the 3175 women with a current screening risk of at least 1/1000 from conventional screening, 2494 (78.6%) women accepted NIPT. Of 934 women with a risk of at least 1/150, 695 (74.4%) accepted NIPT, and 1799 (80.3%) of 2241 with a risk of between 1/151 and 1/1000 accepted NIPT. The average gestation at which NIPT was performed was 14^+2^ weeks. One hundred and ninety three (6%) women were lost to follow-up or miscarried: 129 in the 1/151-1/1000 group and 64 in the higher risk group. Fifty nine (2.4%) results predicted aneuploidy (fig 1); 48 (81%) of these were in pregnancies with risks of at least 1/150, and 11 (19%) were in the 1/151 to 1/1000 group. There were 63 (2.5%) failed and 31 (1.2%) inconclusive results (fig 1). No abnormal results occurred in the failed NIPT group and three in the inconclusive group, two detected by repeat NIPT (one trisomy 21 and one discordant trisomy 18) and the third (trisomy 13) by amniocentesis following detection of sonographic abnormalities.

Forty seven (80%) of the 59 women accepted confirmatory invasive testing, which identified discordant results in five pregnancies (one Down’s syndrome, four trisomy 18), three in the high risk group and two in the intermediate risk group, and confirmed the abnormality in the other 42 pregnancies, 35 of which were terminated and seven continued (fig 1). Two of the remaining 12 women miscarried before making a decision, and 10 (17%) women with a positive NIPT result declined invasive testing. Two of these women had a termination of pregnancy without further testing. Reasons were not documented in one case, and ultrasound strongly indicated trisomy 18 in the second. Eight women continued their pregnancies, with the abnormality confirmed in all eight after birth (fig 1). Of 42 pregnancies with a confirmed positive NIPT result for Down’s syndrome, 13 (31%) of which were continued, 10 live births occurred. The average gestational age at the time invasive testing was offered following a positive NIPT result was 16 weeks for both the high risk and intermediate risk groups, with 16 women having chorionic villus sampling and 31 amniocentesis for confirmation of NIPT results. In the cohort accepting NIPT, the overall sensitivity and specificity for NIPT in detecting aneuploidy, excluding the pregnancies in which no confirmation was available (two miscarriages and two terminations), was 100% (95% confidence interval 95% to 100%) and 99.6% (99.1% to 99.9%) respectively. The positive predictive value was 92% (81% to 97%) overall—94% (83% to 99%) in the high risk group and 82% (48% to 98%) in the intermediate risk group.

One hundred and sixty six (17.8%) women with a standard screening risk of at least 1/150 opted for invasive testing directly. Three (2%) invasive tests failed. The reasons for test failure are unknown; most commonly this is due to poor DNA quality or maternal blood contamination. In two cases, the invasive tests were repeated and found to be abnormal. In the third case, the mother opted for NIPT, which was normal. Fifty eight (35%) women with risks of at least 1/150 who chose direct invasive testing had abnormal results; one miscarried and 55 chose termination (fig 1). There were 29 pregnancies with Down’s syndrome in this group, of which two (7%) continued and resulted in liveborn babies. This is substantially lower than the 13/42 (31%) who continued after an abnormal NIPT result (P=0.0003).

### Uptake

We predicted that 72.5% women with a risk of least 1/150 would accept NIPT and 20% would opt for invasive testing directly. Of all women with a positive NIPT result, 80.4% underwent subsequent confirmatory invasive testing. In units participating in the study, uptake of invasive testing before availability of NIPT was 54% in women with a risk of at least 1/150. Uptake of NIPT was not substantially different in one stop and two stop clinics. In the study, uptake of follow-on testing overall (NIPT and invasive testing combined) in these women at high risk was 92.5%, higher than before availability of NIPT. For the scenario analysis, which does not allow direct invasive testing, the uptake of NIPT was 91%. In women with a lower screening risk (1/151 to 1/1000), the uptake of NIPT was predicted to be 70.5%.

### Costs and consequences of adding contingent NIPT to current Down’s syndrome screening programme

Results were qualitatively similar with both sets of data (national data, RAPID data). Results using national data are described here, with the results using study data in appendix C. Table 3 gives the number of women expected to accept Down’s syndrome screening and subsequent NIPT or invasive testing, potential miscarriages, and the associated costs in a population of 698 500 pregnant women and show the effect of adding NIPT as a contingent test for standard screening risks of at least 1/150, 1/500, or 1/1000 (see also fig 3). Use of NIPT as a contingent test with a risk threshold of 1/150 with the option of direct invasive testing resulted in a non-significant increase in the number of cases of Down’s syndrome detected by 195 (95% uncertainty interval −34 to 480) while requiring 3368 (2279 to 4027) fewer invasive tests and resulting in 17 (7 to 30) fewer procedure related miscarriages for a non-significant reduction in overall total costs (−£46 000, £−1 802 000 to £2 661 000) (table 4). Using the same screening threshold without the option of direct invasive testing, we saw a slightly lower increase in Down’s syndrome cases detected by NIPT, but the reduction in invasive tests, and thus iatrogenic miscarriages, was greater, and the reduction in cost was maintained (tables 3 and 4[Table tbl3 tbl4]; fig 3).

**Table 3 tbl3:** Costs and outcomes of each pathway (in screening population of 698 500 pregnant women), using national data

Testing strategy	Screening				NIPT (£250)			Invasive testing			Total costs (£000)	DS positive		IPD related miscarriage (No)
	No	Test positive	£000		No	£000		Direct No	After NIPT No	£000		NIPT or IPD	Confirmed by IPD	
Current	462 407	10 635	13 312		0	0		5743	0	3733	17 045	577	577	29
NIPT ≥1/1000	462 407	45 316	13 312		32 160	8940		2127	297	1576	23 829	833	732	12
NIPT ≥1/500	462 407	26 357	13 312		18 795	5225		2127	282	1566	20 103	814	719	12
NIPT ≥1/150	462 407	10 635	13 312		7711	2143		2127	248	1544	17 000	772	688	12
NIPT ≥1/1000—no direct IPD	462 407	45 316	13 312		34 128	9487		0	664	432	23 231	826	601	3
NIPT ≥1/500—no direct IPD	462 407	26 357	13 312		20 762	5772		0	649	422	19 506	807	587	3
NIPT ≥1/150—no direct IPD	462 407	10 635	13 312		9678	2690		0	615	400	16 403	765	556	3

**Figure f3:**
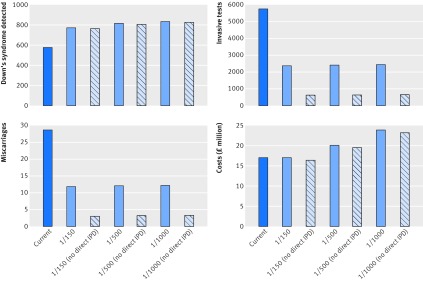
**Fig 3** Benefits and costs of Down’s syndrome screening pathway nationally for current pathway and using non-invasive prenatal testing as contingent test for women with risk of ≥1/150, 1/500, and 1/1000. Estimates are based on population of 698 000

**Table 4 tbl4:** Incremental costs and outcomes compared with current pathway (in screening population of 698 500 pregnant women), using national data

Testing strategy	Per 698 500 pregnant women (95% uncertainty interval)				
	Incremental DS positive NIPT or IPD	Incremental DS confirmed by IPD	IPD avoided	IPD related miscarriage avoided	Incremental costs* (£000)
NIPT ≥1/1000	256 (12 to 529)	155 (−137 to 446)	3319 (2436 to 4252)	16.6 (7.1 to 30.5)	6783 (824 to 17 096)
NIPT ≥1/500	237 (−10 to 557)	141 (−130 to 443)	3334 (2378 to 4225)	16.7 (7.1 to 30.8)	3058 (−482 to 8969)
NIPT ≥1/150	195 (−34 to 480)	111 (−135 to 356)	3368 (2779 to 4027)	16.8 (7.4 to 30.4)	−46 (−1802 to 2661)
NIPT ≥1/1000—no direct IPD	249 (−12 to 511)	24 (−252 to 287)	5079 (4271 to 5901)	25.4 (11.5 to 45.4)	6186 (−210 to 16 983)
NIPT ≥1/500—no direct IPD	230 (−49 to 523)	10 (−254 to 280)	5094 (4273 to 5894)	25.5 (11.7 to 45.9)	2460 (−1565 to 8950)
NIPT ≥1/150—no direct IPD	187 (−52 to 427)	−21 (−253 to 200)	5128 (4405 to 5881)	25.6 (11.7 to 46.2)	−643 (−3025 to 2822)

DS=Down’s syndrome; IPD=invasive prenatal diagnosis; NIPT=non-invasive prenatal testing.

*At NIPT test costs £250.

### Sensitivity analysis

Few parameters appreciably influenced the relative costs of a programme incorporating NIPT compared with current Down’s syndrome screening (moving NIPT from being cost saving to cost neutral; appendix C). At a screening threshold of 1/150 and allowing direct invasive testing, the results of the analysis using national data would change from decreasing costs to increasing costs if the uptake of the current screening programme was 66.0% or lower, the uptake of Down’s syndrome screening with NIPT was 66.4% or higher, or the uptake of NIPT in women with a risk of at least 1/150 was 71.3% or lower.

### Varying uptake

We calculated the incremental costs of implementing NIPT at different uptake levels for NIPT and direct invasive testing in high risk pregnancies. As uptake of NIPT and invasive testing are not independent, we used figures from study centres with the lowest and highest NIPT uptake and the lowest and highest overall uptake for NIPT and direct invasive testing combined (table 5). Using national data, when NIPT uptake was lowest (68.5%), invasive testing uptake was highest (25.9%) and incremental costs increased compared with the main analysis. Conversely, when NIPT uptake was highest (96.6%), direct invasive testing uptake was lowest (3.4%) and incremental costs decreased compared with the main analysis.

**Table 5 tbl5:** Incremental costs compared with current pathway (in screening population of 698 500 pregnant women) for range of uptake values for non-invasive prenatal testing (NIPT) and invasive prenatal diagnosis (IPD) in high risk pregnancies, using national data

Scenario	Uptake (%)				Incremental costs* (£000)	
	NIPT	IPD	Total		1/150	1/150—no direct IPD
Main analysis	72.5	20.0	91.0		−46	−643
Lowest NIPT uptake (Whittington Hospital NHS Trust)	68.5	25.9	94.4		235	−527
Highest NIPT uptake and highest total uptake (Salisbury NHS Foundation Trust)	96.6	3.4	100.0		−427	−337
Lowest total uptake (Barking, Havering and Redbridge University Hospitals NHS Trust)	74.5	13.9	88.5		−404	−728

Uptake figures were derived from table 1 but include trisomy 21 only.

*At NIPT test costs £250.

### Varying cost of NIPT

Some uncertainty surrounds the test cost of NIPT, and the marginal costs of NIPT testing strategies versus current screening are very sensitive to this. We calculated the incremental costs of implementing NIPT at different test costs of NIPT (fig 4) and determined the point at which it would be cost neutral. Using national data, at a screening threshold of 1/150, NIPT would be cost neutral compared with current screening if the cost of NIPT was £256 with the option of direct invasive testing and £316 without this option (appendix C). At a screening threshold of 1/500, the values are £89 and £133; at 1/1000, they are £42 and £71. As the threshold is reduced, the costs of NIPT must be lower and lower to remain cost neutral. Retaining the option of direct invasive testing also necessitates a lower NIPT cost if the programme is to remain cost neutral.

**Figure f4:**
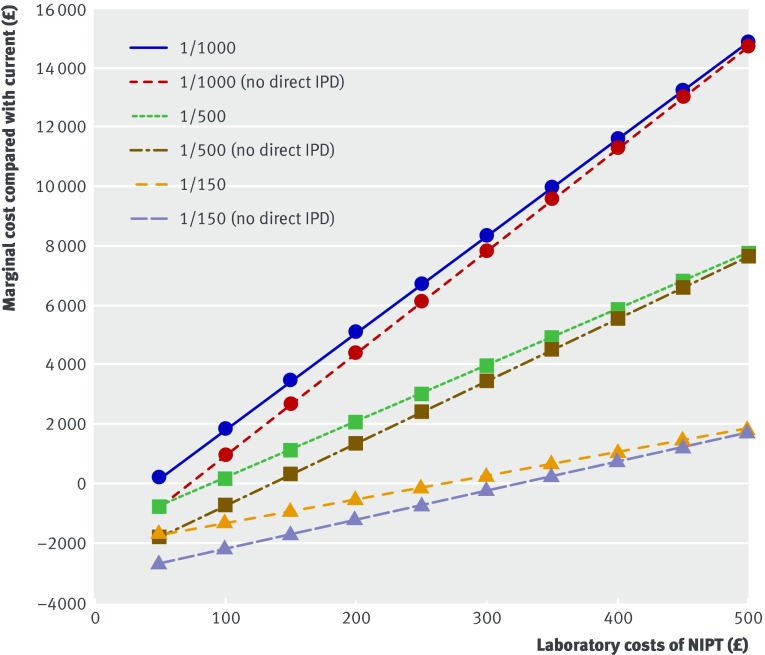
**Fig 4** Marginal costs for different base prices of implementing non-invasive prenatal testing (NIPT) as contingent test for Down’s syndrome for women with screening risk ≥1/150, 1/500, or 1/1000. Dashed lines indicate costs without option of direct invasive prenatal testing (IPD); solid lines indicate costs allowing option of direct IPD without previous NIPT. Figures are based on national data

We re-ran our analysis separately for data from one stop and two stop clinics, and the overall findings did not vary (appendix C).

## Discussion

Our results support the use of NIPT in our public sector Down’s syndrome screening pathway. Implementation as a contingent test at the current risk threshold of 1/150 improves performance by significantly decreasing the false positive rate with a subsequent significant decrease in invasive tests needed and procedure related miscarriages. We found no significant effect on costs and an increase in the number of cases of Down’s syndrome cases detected. Lowering the risk threshold will increase the overall costs, while maintaining a significant reduction in invasive testing and procedure related miscarriages, but with no significant further effect on Down’s syndrome cases detected (fig 3). Increasing the cost of NIPT—if, for example, a licence fee was needed in view of intellectual property considerations—would increase overall costs and alter the cost neutral point (fig 4). Furthermore, if a large proportion of women at high risk opt for direct invasive testing, as has been reported in units with a very high (98.2%) uptake of screening where 38% of high risk women opted for direct invasive testing,^24^ costs would also increase. As uptake of Down’s syndrome screening in our study (76%) was closer to the national average (66%),^25^ we suggest that uptake of direct invasive testing, if NIPT is implemented nationally with this option, is likely to be closer to the figure suggested from our data.

Additional benefits, not captured by the health economic analysis, are linked to offering a test that women welcome as it increases confidence in results and reduces the need for invasive testing.^33^ Notably, approximately one third of women with a confirmed positive NIPT result chose to continue their pregnancy, suggesting that the high uptake of NIPT includes women who would like additional information for preparedness and not necessarily for decision making about termination of pregnancy. Although the numbers are small, this finding indicates that the birth rate of infants with Down’s syndrome may not change significantly if NIPT is introduced more widely, an observation in keeping with two regional US studies suggesting that NIPT has not affected the number of infants born with Down’s syndrome,^4^ ^41^ as well as one UK study that also showed that some women continued the pregnancy with a diagnosis of Down’s syndrome after NIPT.^24^ In addition, NIPT can clearly be offered in the public sector without compromising informed choice. Analysis of decision making in a subset of women offered NIPT in our study (n=585) showed that rates of informed choice were high (89%),^42^ but this may reflect the effort made to educate all health professionals before the start of the study and the increased time spent counselling women.^32^ Further assessment is therefore needed to explore whether this high rate of informed choice can be maintained outside a research setting.^42^

The outcomes presented here, derived from uptake and outcome data collected in the RAPID study that are then modelled to reflect the effect nationally, are similar to those predicted from hypothetical scenarios using entirely modelled data,^9^ except that offering NIPT to women with risks of 1/150 or greater increases rather than decreases detection of Down’s syndrome when modelled data are used. This is a reflection of the increased uptake (>90%) of further testing (NIPT or direct invasive testing) in the NIPT pathways compared with the current pathway (54%), thus increasing detection of Down’s syndrome cases in the 1/150 or greater group as NIPT has a very high sensitivity.

Procedure related miscarriages are further decreased if invasive testing is offered only after a positive NIPT result. However, many people advocate allowing direct access to invasive testing for women at very high risk or those with abnormal ultrasound findings.^26^ Of note, in women with a risk of at least 1/150, 58 (34.9%) abnormalities were seen in the 166 women choosing direct invasive testing compared with 45/695 (6.5%) in those choosing NIPT, reflecting the fact that those choosing direct invasive testing are at higher risk, an average of 1/8 compared with the average risk of 1/30 in those choosing NIPT. In the study. NIPT was offered to women with a screening result of at least 1/1000, allowing evaluation of behaviour relating to choice and the effect of using lower risk thresholds of 1/500 and 1/1000 to show that these would lead to slightly more positive results but increased costs compared with the current pathway. For introduction of NIPT to be cost neutral in these scenarios, the laboratory cost of NIPT would need to fall substantially (fig 3).

### Strengths and limitations of study

The main strength of our analysis was that we used actual clinical data, collected in a fully funded public sector maternity care system in units with a range of screening uptakes and modes of service delivery. These results thus reflect women’s behaviour in real life regarding uptake of Down’s syndrome screening, NIPT, and invasive testing as inputs for our model.

Limitations include the fact that data might not be nationally representative as they were collected in only eight hospitals. The proportion of women with a Down’s syndrome screening risk of at least 1/150 was higher than the current national figure, and the number of women with a risk between 1/151 and 1/500 or 1/1000 was lower. However, re-analysis using age standardised study data and national Down’s syndrome screening figures showed similar trends. Furthermore, uptake of Down’s syndrome screening is around 66% nationally,^25^ compared with >75% in study clinics before the start of the study. In addition, the uptake of screening during the study fell slightly overall, possibly because women accessed commercial NIPT and subsequently declined screening when booking for NHS maternity care. As a result, uncertainty exists about whether introducing NIPT would change screening uptake, so we assumed that it was unchanged. Furthermore, as women with a risk of at least 1/150 could opt directly for invasive testing without undergoing NIPT first, we assumed that they would accept NIPT if this was the only available option. Thus we believe that our estimate based on women undergoing further testing (NIPT or direct invasive testing) is representative for the uptake of NIPT in this scenario. These findings are relevant to settings with well established Down’s syndrome screening programmes, as in the United Kingdom. For countries where these are not established, where attitudes to screening and diagnosis vary, or where the geography is such that women do not have access to expert ultrasonography, alternative implementation strategies may be more appropriate.^5^ ^43^ ^44^

A further limitation of the economic analysis is the uncertainty surrounding the cost of NIPT. To overcome this, we present results for a range of values, taking as our base case a value of £250, which is the cost in our laboratory. Our economic analysis takes a narrower costing perspective than one that is commonly used in UK economic evaluations (NHS and personal social services perspective), reflecting the costs incurred by the UK National Screening Committee, which delivers the Down’s syndrome screening programme and which will decide whether to implement NIPT. Finally, NIPT can detect trisomies 13 and 18, but we have not analysed this in detail in our study, as screening for these conditions was not yet implemented in all participating units. Further evaluation in a larger population will be needed to draw meaningful conclusions, but most of these other trisomies (23/35) in the study occurred in those women at high risk who opted directly for invasive testing. This is probably because these conditions are usually associated with other sonographic anomalies.^45^ Of note, other chromosomal rearrangements, including the case of triploidy in our study, will not be detected using the method we report and women must be made aware of that limitation, particularly in the presence of fetal sonographic abnormalities.^46^

### Conclusions

Using empirical data on uptake of testing, we have shown that NIPT can be provided effectively, and without increasing costs, as part of a publicly funded national Down’s syndrome screening pathway with the provision of NIPT testing in a public sector laboratory. In view of the small number of discordant results, we concur with other authors that this has to be considered a highly sensitive screening test and that invasive testing will be needed for confirmation. We have also shown that some women will use NIPT for information only to prepare themselves for the birth of a baby with Down’s syndrome, so maternity services must have coordinated pathways for these families, including close monitoring in late pregnancy, and Down’s syndrome live birth rates may not change significantly. We conclude that a strong case exists for implementation of NIPT as part of the Down’s syndrome screening programme to improve the quality of care for pregnant women and the performance of the programme as a whole. These data were presented to the UK National Screening Committee as part of its evidence review. The committee considered our findings before going to public consultation on its recommendation for NHS implementation of NIPT as a contingent test for all women with a traditional Down’s syndrome screening risk of at least 1/150. Subsequent to this consultation, the committee has recommended to the government that NIPT be implemented and is now awaiting ministerial decisions.

**What is already known on this topic**

Non-invasive prenatal testing (NIPT) based on analysis of cell-free DNA in maternal plasma is a highly accurate screening test for Down’s syndrome that is likely to be cost effective if offered as a contingent screening test
Although NIPT is increasingly available worldwide, access is primarily through the private sector
The costs and consequences of implementation of NIPT into public sector maternity care have not been assessed in a clinical setting

**What this study adds**

NIPT can be provided effectively as part of a publicly funded national Down’s syndrome screening pathway
It will improve the performance of the programme without significantly increasing costs if offered as a contingent test at a risk threshold of 1/150
Uptake of NIPT by women is high, with some of them seeking information to prepare for the birth of a baby with Down’s syndrome

## Web Extra

Extra material supplied by the author

## Notes

We are grateful to the parents who participated in the study. We also thank the health professionals at each of the participating units for their help with the study.

Contributors: LSC conceived the study, obtained the funding for it, and was responsible for the study overall. LSC and SM designed the study and managed the research process. LSC, SM, TIV, and DW did the data analysis. RD led the study set-up and was responsible for the day to day supervision of study sites, data collection, and data management. CL assisted with study set-up and data collection. FMcK, SM, and LJ were responsible for laboratory analysis. MH assisted with study design, study set-up, and ethical approvals. AH was involved in data collection and management. MK, AMcE, and JF assisted with study design. LSC, SM, TIV, DW, RD, LJ, MH, LC, MK, AMcE, and JF were members of the Study Steering Committee. LSC wrote the first draft of the manuscript, and all authors contributed to revising and editing the manuscript. LSC is the guarantor.

Ethical approval: National research ethics approval (13/LO/0082) was obtained. All participants gave written consent.

Data sharing: No additional data available.

Transparency declaration: The lead author affirms that the manuscript is an honest, accurate, and transparent account of the study being reported; that no important aspects of the study have been omitted; and that any discrepancies from the study as planned (and, if relevant, registered) have been explained.
