# Supplementary material for: Uptake, outcomes, and costs of implementing non-invasive prenatal testing for Down’s syndrome into NHS maternity care: prospective cohort study in eight diverse maternity units
Source: BMJ. 2016 Jul 4;354:i3426. doi: 10.1136/bmj.i3426 (PMC4933930; doi:10.1136/bmj.i3426)
Supplement: Supplementary file 1 — Appendix A [file chil030961.ww1_default.pdf]

Patient information sheet – Booking
Study title: An evaluation of NIPT for aneuploidy in an NHS setting
New tests for detecting Down's syndrome in pregnancy

Principal Investigator: Professor Lyn Chitty, Professor and Consultant in Genetics and Fetal Medicine

We would like to let you know about a new research study. Before you decide whether or not to participate we would like you to understand why the research is being done and what it would involve for you. This information will be discussed with you when your midwife is explaining the blood tests that are offered at booking. Please ask if anything is not clear and take time to decide whether or not you wish to take part.

What is the purpose of the study?

The aim of this study is to develop better and safer ways of detecting Down's syndrome and some other chromosomal conditions in pregnancy.

What is Down's syndrome?

Down's syndrome is a life-long condition that causes delays in learning and development as well as some physical problems. Down's syndrome is caused by an extra copy of chromosome 21. Chromosomes are present in almost all human cells and store our genetic information. People usually have 46 chromosomes in each cell, but occasionally extra copies of chromosomes can be present. Other conditions resulting from a change in the number of chromosomes are Edward's syndrome (extra copy of chromosome 18),Patau syndrome (extra copy of chromosome 13) and Turner syndrome.

What are the current tests for Down's syndrome?

Screening tests for Down's syndrome are currently offered to all pregnant mothers in England. These tests use a blood sample taken from the mother or measurements taken from ultrasound scans or both to work out the chance that the baby will have Down's syndrome. These tests cannot tell us for sure if a baby has Down's syndrome but they can tell us how likely it is that a baby has Down's syndrome. For example, the result "1 in 100" means that there is one chance in 100 that your baby has Down's syndrome. If your screening tests shows that you have a higher chance (results between 1 in 2 and 1 in 150) of having a baby with Down's syndrome you will be offered a diagnostic test that will give you a clear yes / no answer. Currently two types of diagnostic tests are available; chorionic villus sampling (CVS) or amniocenteses. These tests are referred to as invasive tests as a needle must be passed through the mother's abdomen to obtain the sample from either the placenta (CVS) or the fluid surrounding the baby (amniocentesis). These tests carry a small risk of miscarriage (in the UK up to 1%). For more information on the tests for Down's syndrome that are available see the NHS "Testing for Down's syndrome in pregnancy" leaflet.

What are the new tests for Down's syndrome?

A new type of blood test for Down's syndrome has been developed, it is called non-invasive prenatal testing (NIPT). This test looks directly at DNA from the baby which we now know is present in the mothers blood during pregnancy. As the blood is taken from the mother's arm, it does not carry a risk of miscarriage. As chromosomes are made of DNA we are able to detect chromosomal abnormalities such as Down's syndrome, Edward's syndrome, Patau syndrome or Turner syndrome by analysing the baby's DNA that is in the mother's blood. Research has shown that NIPT is much more accurate than current screening tests and will detect around 98% of cases where the baby has Down's syndrome. However, there is a small chance (around 0.3%) it could falsely indicate that the baby has Down's syndrome. For this reason if you have a predicted to be affected NIPT result you will be offered an invasive test to confirm the result.

In addition, we would like to improve the current Down's syndrome screening test by looking for additional factors in the mother's blood sample that could improve the detection of Down's syndrome and may also help identify pregnancies at high risk of other adverse pregnancy outcomes (pre-eclampsia and intra-uterine fetal growth restriction). This will also hopefully help us to more accurately identify which women should be offered further testing.

How will NIPT be offered in the research study?

In this research study we would like to work out the best way of offering NIPT within the NHS. As the test is expensive it cannot be offered to every pregnant mother. So all pregnant women will continue to be offered the current screening tests and only those that have a screening result of "1 in 1000" or higher will be offered the NIPT test. Mothers who have a result of "1 in 150" or higher will be offered an invasive test as is current practice, but they will also be offered NIPT. This approach will allow us to detect some babies with Down's syndrome that might be missed with current screening and for those with a highly unlikely to be affected result it will offer greater reassurance. This approach will also mean that fewer women will be offered an invasive test with its associated risk of miscarriage.

Your midwife will explain the various tests for Down's syndrome that are available when she discusses the other blood tests at your booking appointment. It is entirely your decision whether or not you want Down's syndrome testing and whether or not you want to be part of the research study.

Why am I being told about the study?

We are telling all pregnant women over 16 years of age attending the unit about the study.

Do I have to take part?

It is up to you to decide whether or not to take part in the research study. If you decide to take part you are free to withdraw at any time without giving a reason. This would not affect the standard of care you receive.

What will happen to me if I take part?

Women who decide to have Down's syndrome screening will be asked if they want to take part in the study. For this part of our study we are asking some women if we can use any excess blood left over from your Down's syndrome screening test, and collect some data related to your pregnancy outcomes. The Down's syndrome screening test will be conducted as normal and you will not receive any additional results from the research. We also need to find out the outcome of your pregnancy. We will try and do this by reviewing your maternity records but we may need to contact you directly.

After you receive your Down's syndrome screening results we may talk to you about the NIPT test and women who chose to participate in this part of the study and have NIPT will be asked to sign a consent form and to give a blood sample which will be used for NIPT. You will be told the results of your NIPT test within 7-10 working days. In addition, as part of the study we would like to gather the views and experiences of women being offered NIPT and we will ask your permission to contact you for an interview or to complete a questionnaire. You will not be eligible for NIPT if you have a multiple pregnancy.

- If your Down's syndrome screening result is lower than 1 in 1000 (so from 1 in 1001 onwards) you will not be contacted about NIPT and your pregnancy care will continue as usual.
- If your Down's syndrome screening result is between 1 in 151 and 1 in 1000 a midwife will speak to you about NIPT.
- If your Down's syndrome screening result is between 1 in 2 and 1 in 150 (the current cut-off for offering invasive testing) a midwife will speak to you about having NIPT, but she will also discuss having an invasive test.

It is important to understand that it will be up to you to decide whether or not to have any of these tests at the time they are offered to you. You do not have to decide now.

When will I get the NIPT result?

You will be phoned with the results of NIPT within 7-10 working days of having the blood test. If you receive a predicted to be affected NIPT result you will be offered further counselling and an invasive diagnostic test if you want it.

Will my taking part in this study be kept confidential?

Yes. We will follow ethical and legal guidelines and all information will be handled in confidence.

What are the possible benefits of taking part?

If you do agree to take part in the study you may benefit from having NIPT for Down's syndrome as including this test in the care pathway means fewer women will need an invasive test, which carries a 1% risk of miscarriage, but we may detect more cases of Down's syndrome. We hope that the results of the study overall will enable us to improve antenatal care provided to mothers by working out the best way to offer NIPT. There will be no financial benefit from participating in the study.

What are the possible disadvantages and risks of taking part?

As NIPT is a blood test, some people may experience bruising where the needle goes in which will disappear over a few days. Making decisions about what tests to have during pregnancy can be difficult and can cause anxiety. It may help to speak to your midwife, family and friends or you can contact the Antenatal Results and Choices (ARC) helpline. Phone: 020 7713 7486

What will happen if I don't want to continue in the study?

You are free to withdraw at anytime. This will not affect your care in anyway.

What will happen to the results of the research study?

The results from our project will be published as research papers in medical journals. No information will be published that will allow you as individual to be identified.

Where can I get further information or discuss any problems?

Please contact a member of the research team on [Insert phone number] to discuss any questions or worries about the study, or if you have any complaints.

If you would like to speak to someone outside your healthcare team for information and support around prenatal testing you can contact the Antenatal Results and Choices (ARC) helpline. Phone: 020 7713 7486

Please contact Patient Advisory Liaison Services (PALS) if you have any concerns regarding the care you have received, or as an initial point of contact if you have a complaint. You can phone PALS on [Insert phone number] or email [Insert email address], you can also visit PALS by asking at any hospital reception.

Who is organising and funding the research?

This research is organised by Research Teams at Great Ormond Street Hospital, University College London Hospital and Salisbury District Hospital. The study is funded by the National Institute for Health Research. A commercial company is covering the cost of the study to improve the current Down's syndrome screening test. The company has no influence on study design or publication of results.

Who has reviewed the study?

All research in the NHS is looked at by independent group of people, called a Research Ethics Committee, to protect your interests. This study has been reviewed and given favourable opinion by the Camden and Islington National Research Ethics Service Committee.

Thank you for taking the time to read this information leaflet.
