# Supplementary material for: Uptake, outcomes, and costs of implementing non-invasive prenatal testing for Down’s syndrome into NHS maternity care: prospective cohort study in eight diverse maternity units
Source: BMJ. 2016 Jul 4;354:i3426. doi: 10.1136/bmj.i3426 (PMC4933930; doi:10.1136/bmj.i3426)
Supplement: Supplementary file 2 — Appendix B [file chil030961.ww2_default.pdf]

Patient information sheet – NIPT

Study title: An evaluation of NIPT for aneuploidy in an NHS setting
New tests for detecting Down's syndrome in pregnancy

Principal Investigator: Professor Lyn Chitty, Professor and Consultant in Genetics and Fetal Medicine

You are being invited to take part in a research study. Before you decide it is important for you to understand why the research is being done and what it will involve. Please take time to read the information carefully and discuss it with others if you wish. Please ask if there is anything that is not clear and take time to decide whether or not you wish to take participate.

What is the purpose of the study?

When you were offered Down's syndrome screening you were also told about a new type of blood test for Down's syndrome that has been developed called non-invasive prenatal testing (NIPT). As this is a new test we are now performing a research study to find the best way of offering NIPT to mothers, to find out what parents and health professionals think of the new test and how it is offered, and to confirm its accuracy.

What is NIPT?

NIPT is done through a blood test from the mother's arm after 10 weeks of pregnancy and like the screening test you have had, it can tell you the chance that the baby has Down's syndrome, Edward's syndrome, Patau syndrome or Turner syndrome. NIPT is much more accurate than current screening tests as it will detect around 98% of Down's syndrome cases. Please see the patient information leaflet "NIPT – a new test for Down's syndrome" for more information.

Why am I being invited to take part in the study?

We are inviting all women with a singleton pregnancy over 16 years of age with a Down's syndrome screening result between 1 in 2 and 1 in 1000 to take part in the study.

What will happen to me if I take part?

If you do decide to take part in the research study you will be given this information sheet and your signed consent form to keep and the midwife will write in your notes that you are part of the research study. A midwife will discuss NIPT with you and offer you this test. How NIPT is offered to you will depend on your screening result as outlined below:

1. **<u>My screening result is between 1 in 2 and 1 in 150:</u>**

If your screening tests shows that you have a risk between 1 in 2 and 1 in 150, this is considered a higher risk result and you would normally be offered an invasive diagnostic test (an amniocentesis or CVS) that will give you a clear yes / no answer as to whether the baby has Down's syndrome (or one of the rarer chromosomal conditions – Edwards syndrome and Patau syndrome). These tests carry a small risk of miscarriage (in the UK up to 1%).

If you choose to take part in the research you will have the option to have NIPT before deciding if you want to have an invasive test. There are four possible results of NIPT:

- **Highly unlikely to be affected result:** As NIPT is more accurate than current screening tests it may mean that your baby is now highly unlikely to be affected with Down's syndrome, Edward's syndrome, Patau syndrome or Turner syndrome. If this is the case you may choose to have an invasive test to confirm the result or you may decide you don't want to have an invasive test. If some abnormalities were seen on your ultrasound scan your doctor may discuss having an invasive test as well, as occasionally there are some rarer chromosomal changes that will not be seen by NIPT but will be seen by invasive testing.
- **Predicted to be affected result:** It is very likely that the baby is affected with Down's syndrome, Edward's syndrome, Patau syndrome or Turner syndrome. You will be offered an invasive test to confirm this result. This is because very occasionally, in 0.3% of cases, NIPT may be incorrect.
- **Inconclusive result**: As this is a new test we occasionally get an inconclusive or 'unclear' result (0.5-4% of cases). If we cannot detect a clear result we will inform you and offer you an invasive test.
- **Failed result**: Occasionally we get a failed result, usually because of a problem in the laboratory, and will offer you a repeat NIPT or an invasive test.

If you would prefer not to have any further testing or would like to have an invasive test and not have NIPT this is entirely your decision and will not in any way affect the care you receive.

2. **<u>My screening result is between 1 in 151 and 1 in 1000:</u>**

In current practice this result is considered a low risk result and you would not normally be offered any further testing. Because NIPT is more accurate than current screening tests we would like to offer the test to as many women as possible, however, it is currently too expensive to offer to all pregnant women. We have decided to offer the test to women with a Down's syndrome screening result between 1 in 151 and 1 in 1000. By doing this it is likely that we will detect a few babies with Down's syndrome that might be missed with current screening, and for those with a highly unlikely to be affected result it will offer greater reassurance. We hope that this will benefit a greater number of mothers.

If you choose to take part in the research you will have NIPT. There are four possible results:

- **Highly unlikely to be affected result:** Your baby is highly unlikely to be affecetd with Down's syndrome, Edwards syndrome, Patau syndrome or Turner syndrome. You will continue with your normal care.

- **Predicted to be affected result:** As this test is more accurate it may detect cases of Down's syndrome that have been missed on the first screening test. It is very likely that the baby is affected with Down's syndrome, Edward's syndrome, Patau syndrome or Turner syndrome. You will be offered an invasive test to confirm this result. This is offered because very occasionally in 0.3% of cases NIPT may be incorrect.
- **Inconclusive result:** As this is a new test we occasionally get an inconclusive or 'unclear' result (0.5-4% of cases). If we cannot detect a clear result we will inform you and offer you another NIPT test.
- **Failed result**: Occasionally we get a failed result, usually because of a laboratory problem, and will offer you a repeat NIPT.

If you would prefer not to have NIPT this is your decision and it will not in any way affect the care you recieve.

The NIPT samples from women who also had an ultrasound abnormality or NT measurement of ≥3.5mm will be investigated further for other chromosomal rearrangements. No results from these investigations will be available during pregnancy or reported as this is a new area of NIPT development.

How is NIPT performed?

If you do decide to take part in the research study you will be given this information sheet and have NIPT, the midwife will take two tubes of blood (20-30ml) that will be sent to the laboratory to test for Down's syndrome, Edward's syndrome, Patau syndrome and Turner syndrome. Testing will be done in Regional Genetics Laboratories in the UK, but samples may also be sent to other laboratories, including commercial companies, and may be sent outside the UK in order to compare different methods of testing. Your sample will be destroyed at the end of your pregnancy.

When will I receive my NIPT results?

You will be called by a midwife with your results within 7-10 working days. If you receive a predicted to be affected NIPT result you will be offered further counselling and an invasive diagnostic test if you want it.

What else might I be asked to do as part of the study?

As part of the study we would like to gather the views and experiences of women being offered NIPT as well as your thoughts on the information provided and we will ask your permission to contact you for an interview or to complete a questionnaire. Further details about these studies will be given at the time we contact you.

Do I have to take part?

It is up to you to decide whether or not to take part in the research study. If you agree to take part, the midwife will give you a consent form to sign. If you decide to take part you are free to withdraw at any time without giving a reason. This would not affect the standard of care you receive.

Will my taking part in this study be kept confidential?

Yes. We will follow ethical and legal guidelines and all information will be handled in confidence.

What are the possible benefits of taking part?

If you do agree to take part in the study you may benefit from having NIPT for Down's syndrome as fewer women will need an invasive test, which carries a 1% risk of miscarriage, and we may detect more cases of Down's syndrome overall. We hope that the results of the study will enable us to improve antenatal care for women by working out the best way to offer NIPT. There will be no financial benefit from participating in the study.

What are the possible disadvantages and risks of taking part?

As NIPT is a blood test, some people may experience bruising where the needle goes in which will disappear over a few days. Making decisions about what tests to have during pregnancy can be difficult and can cause anxiety. It may help to speak to your midwife, to your family and friends or you can contact the Antenatal Results and Choices helpline. Phone: 0207 7137486

What will happen if I don't want to continue in the study?

You are free to withdraw at anytime. This will not affect your care in anyway.

What will happen to the results of the research study?

The results from our project will be published as research papers in medical journals. No information will be published that will allow you as an individual to be identified.

Where can I get further information or discuss any problems?

Please contact a member of the research team [Insert research midwife name] on [Insert phone number] to discuss any questions or worries about the study, or if you have any complaints.

If you would like to speak to someone outside your health care team for information and support around prenatal testing you can contact the Antenatal Results and Choices (ARC) helpline. Phone: 020 7713 7486

Please contact Patient Advisory Liaison Services (PALS) if you have any concerns regarding the care you have received, or as an initial point of contact if you have a complaint. You can phone PALS on [Insert phone number] or email [Insert email address], you can also visit PALS by asking at any hospital reception.

Who is organising and funding the research?

This research is organised by Research Teams at Great Ormond Street Hospital, University College London Hospital and Salisbury District Hospital. The study is funded by the National Institute for Health Research.

Who has reviewed the study?

All research in the NHS is looked at by independent group of people, called a Research Ethics Committee (REC), to protect your interests. This study has been reviewed and given favourable opinion by the Camden and Islington National REC.

Thank you for taking the time to read this information leaflet.
