# Supplementary material for: Uptake, outcomes, and costs of implementing non-invasive prenatal testing for Down’s syndrome into NHS maternity care: prospective cohort study in eight diverse maternity units
Source: BMJ. 2016 Jul 4;354:i3426. doi: 10.1136/bmj.i3426 (PMC4933930; doi:10.1136/bmj.i3426)

Online supplementary material: cost consequences analysis

Literature review

Several studies have examined the costs and benefits of NIPT for Down syndrome screening [1-13]. Several of these studies focused on the USA [3, 4, 7, 10], Australia [1, 9] or Canada [8]. Two studies focused on the UK [5, 12], one on the Netherlands [2] and one on Belgium [6].

Palomaki *et al.* found that compared with current screening, NIPT contingent testing would detect slightly fewer cases, and that fewer invasive tests would be performed, fewer procedure related miscarriages would occur, and costs would be lower [10]. The analysis however did not include costs of NIPT, which may be substantial. Garfield & Armstrong found that NIPT contingent screening in high risk women would be more effective in terms of procedure-related miscarriages and Down syndrome cases detected and cost saving compared to the current screening programme when the costs of NIPT were no higher than $1200 [4]. Cuckle *et al.* compared different NIPT pathways with the current pathway and showed that the total costs would increase significantly if NIPT was used as a first line test [3] and therefore NIPT as a contingent test was recommended. In another study from the USA, the cost-effectiveness of NIPT contingent testing compared with NIPT as a diagnostic test in high risk women was assessed [7]. NIPT as contingent testing was cost-effective compared to NIPT as a diagnostic test, but the authors did not investigate the cost-effectiveness of NIPT compared to the current screening programme, making it difficult to assess if NIPT contingent testing was cost-effective. O'Leary *et al.* demonstrated that NIPT contingent testing in high risk women would increase the costs of screening in Australia, and that as long as the uptake of NIPT was higher than the uptake of invasive testing in the current pathway, more cases would be detected with NIPT [9]. Song *et al.* found that offering NIPT to women at high risk of a Down syndrome pregnancy would increase the detection of Down syndrome cases and decrease the total health care costs [11]. The authors included lifetime costs of caring for a child with Down syndrome, which had a large influence on the results. NIPT would lead to increased costs if these costs were not included. Wald and Bestwick investigated the detection rate and genetic test costs of a protocol combining screening and NIPT in the UK, but did not include any costs for invasive testing [12]. They concluded that their protocol would be cheaper than universal DNA testing.

Ayres *et al.* studied the consequences of replacing the current screening protocol with several different NIPT pathways (first line testing or contingent testing with or without first line NIPT for certain high risk women) [1]. They concluded that NIPT contingent screening was cost saving, but less effective; first line testing was more effective and more costly. The most cost-effective option was NIPT contingent screening with NIPT as first line test for women >40 years. In a Dutch study, the current screening programme was also compared with first line as well as contingent NIPT testing [2]. Both NIPT strategies increased the detection of Down syndrome cases at higher total costs. The authors reported that the costs of the programme would be unacceptably high if NIPT were implemented as a first line test, and hence the NIPT contingent pathway led to more favourable results. A similar comparison was performed by Morris *et al.,* Neyt *et al.* and Okun *et al.*, all concluding that first line testing was unlikely to be cost-effective and showing more favourable

results for NIPT contingent testing [5, 6, 8]. In the study by Morris *et al.* NIPT contingent testing could decrease the procedure related miscarriages without increasing the total costs of the screening pathway [5]. The authors looked at different screening risk thresholds for offering NIPT as a contingent test. This threshold value had a large influence on the results, although the detection of Down syndrome cases was lower than in the current programme for each threshold because of assumptions made surrounding the uptake of testing. First line testing was calculated to detect more cases, but at significantly increased total costs of the screening programme. In the study by Walker *et al.* first line NIPT testing was cost saving and more effective compared to the integrated test from a societal perspective (including lifetime costs of a child with Down syndrome) [13]. However, NIPT was more costly when a narrower perspective was taken, not including lifetime costs of a child with DS.

Influence of uptake and cost of NIPT

Based on the information from the studies described above, it is highly unlikely that first line NIPT testing would be cost-effective, unless the costs of NIPT were to fall dramatically. On the other hand, NIPT as contingent testing may have the potential to increase the detection of Down syndrome cases and/or decrease the number of procedure related miscarriages, without increasing costs. In most studies, assumptions regarding the uptake of NIPT were made, because of a lack of empirical data, e.g., assuming 100% uptake or assuming that uptake would be the same as for invasive testing. These assumptions are important because both the number of cases detected and total costs will depend on NIPT uptake, and therefore there is considerable uncertainty about the cost-effectiveness of NIPT due to uncertainty about the uptake of the test. Another area of uncertainty is the cost of NIPT, especially in the NHS since it is not routinely provided at present. In the private sector in the UK, prices for NIPT vary from £400-£900 [5]. In summary, there is little economic evidence for NIPT that is directly relevant to the UK NHS (screening practices, costs and uptake are likely to vary between countries), and what evidence there is has been required to make simplifying assumptions about the uptake of testing.

Morris *et al.* showed that if NIPT could be offered to more women (for example using a screening risk cut-off of 1/2000), more cases would be detected than with a screening risk cut-off of 1/150, but at increased costs[5]. It is possible that the costs of NIPT contingent screening relative to the current DS screening pathway will vary by screening cut-off, since this affects costs and outcomes. It is therefore useful to investigate the effects of NIPT testing at different screening risk cut-offs to find the best strategy without increasing costs.

Limitations of QALYs

In the UK the recommended outcomes for economic evaluations are quality-adjusted life years (QALYs), and the recommended cost-effectiveness measure is based on the incremental costs per QALY gained [14]. One advantage of using the incremental costs per QALY gained is that there is a published threshold that can be used to judge whether or not an intervention is good value for money (£20,000 to £30,000 per QALY gained [14]). However, measuring QALYs arising from prenatal testing is challenging. Only one study has reported the QALY gains in the context of NIPT testing for Down syndrome [7]. The authors assumed that women would have a lower quality of life during their remaining years of life after giving birth to a baby with Down syndrome, during 2 years after

miscarriage or termination and during 1 year after a false positive result. It is difficult to determine the quality of life of a child with Down syndrome and of other unaffected children as well as the quality of life of the parents. There is also uncertainty about what happens when a pregnant mother decides to terminate a Down syndrome affected pregnancy. She might get pregnant again and have another (healthy) baby or not. Next to this, some women might decide not to terminate the pregnancy in case of a Down syndrome affected fetus, but use the information obtained from screening to prepare for the birth of an affected baby. Having this information and being able to prepare for an affected baby, or the reassurance that the baby is unaffected, could also increase the quality of life of the parents. Due to these difficulties, the economic consequences of prenatal testing are not usually reported using QALYs.

As in previous studies, we report instead the number of Down syndrome cases detected, the number of procedure-related miscarriages and costs. Our measure of costs is the combined cost of Down syndrome screening, NIPT and invasive diagnostic testing; it does not include the costs of pregnancy outcomes nor of costs incurred beyond birth, such as lifetime costs of caring for a baby with Down syndrome, according to the NSC's preferred costing methodology. .

Cost consequences analysis of NIPT for Down syndrome as contingent testing compared with the current DS screening programme in the UK National Health Service

Methods

Model structure

A decision tree was developed to assess the costs and outcomes of 4 different screening pathways:
(1) the current pathway;
(2) NIPT as contingent screening for women with a ≥1/150 risk;
(3) NIPT as contingent screening for women with a ≥1/500 risk; and
(4) NIPT as contingent screening for women with a ≥1/1000 risk.

Current pathway

Figure 2 in the main paper depicts the current screening pathway and the NIPT contingent screening pathway. Briefly, in the current screening pathway, women are offered invasive testing when their risk based on the combined or quadruple test is ≥1/150. There is a small risk of a procedure related miscarriage, so some high risk women decide not to undergo any further testing. If the result of the invasive test is positive, women can decide to terminate the pregnancy.

RAPID study pathway

In the NIPT pathway, women are offered a NIPT test after a high risk result (depending on the threshold) based on the combined or quadruple test. The pathways for the different thresholds are all similar except for the threshold risk at which NIPT is offered as contingent screening (≥1/150, ≥1/500 or ≥1/1000). If the NIPT test result is positive, an invasive test is offered to confirm the diagnosis. Some women with a risk ≥1/150 after the combined or quadruple test might decide to have an invasive test directly and not have NIPT first. We also assessed the costs and outcomes of

modifying the NIPT contingent screening pathway as described to preclude the option of having an invasive test directly after screening, so that only women with a positive NIPT result were offered invasive testing.

Focus on Down syndrome screening

While NIPT may have a role in detecting other trisomies, the over-arching aim of the RAPID evaluation study is to evaluate NIPT for Down syndrome. For the economic analysis we therefore focused on the Down's syndrome screening pathway and did not include other trisomies, even though women were offered testing for trisomies 13 or 18 in some clinics. For the present analysis, we categorised women based on Down syndrome risk only and we excluded any testing performed solely based on trisomy 13 or 18 risks. Six women with a Down syndrome risk between 1/151 and 1/1000 underwent invasive testing before NIPT because of a high risk of trisomy 13 or T18. These invasive tests were excluded from the present analysis, as we assumed that only women with a Down syndrome risk ≥1/150 could undergo invasive testing directly after screening. Three of these women also accepted NIPT, the other three women declined NIPT. We therefore assumed that for the DS screening pathway, the first three woman would undergo NIPT and no subsequent invasive test and the other three woman would undergo no further testing after screening.

Model inputs

We ran our analyses using two datasets; the first was based predominantly on data from the RAPID evaluation study, supplemented with national data where necessary; the second was based predominantly on national data, using data from the RAPID evaluation study mainly to quantify NIPT uptake behaviours. Data from the RAPID evaluation study were used to assess the uptake of NIPT contingent screening and the uptake of subsequent NIPT and invasive testing. Data from the RAPID study were also available for the different outcomes of the tests and pregnancy outcomes. The uptake of screening and invasive testing in the current pathway was assessed using a historical dataset collected in the same clinics as the RAPID data, but before introduction of NIPT, during the period 2011 to 2012. The key parameters of the model, and the sources of these data, are listed in Table 2 (main paper). The uptake of screening in the eight clinics involved in the RAPID study was slightly higher before NIPT was offered than during the RAPID study (76.0 vs 78.7%). This might be caused by the fact that some women undergo NIPT in the private sector instead of undergoing screening in the national programme. In the present analysis we assumed that this decrease in uptake of screening is unrelated to the RAPID study, so we used the same uptake value as in the current programme (78.7%). Our analysis considers different Down syndrome screening cut-offs and we require NIPT uptake figures for these cut-offs. The uptake of NIPT was 70.5% in women with a risk between 1/150 and 1/1000. This percentage was 72.5 for women with a high risk result. More than 80% of the women with a positive NIPT result underwent invasive testing to confirm the diagnosis, while 54% of the women with a high risk result in the current screening programme underwent invasive testing.

The number of women with a serum screen risk of ≥1/150 was higher than the national figure. In the analysis using the RAPID data, screening results were age-standardised to make them more representative of the national screening programme. For some input parameters, no data was available from the RAPID study (for example fetal loss after invasive testing or the incidence of Down

syndrome in the general population) and national sources were used instead. No national data is available yet on any outcomes related to NIPT (e.g., uptake of NIPT, number of positive results), so for the analysis based on national sources, we used RAPID data for these input parameters. In the RAPID study, five of the positive NIPT results were found to be false-positives (9.1%), and the positive predictive value of NIPT has been found to be less than 100% in several other studies. For the national dataset, we therefore assumed a positive predictive value of 90.9% and varied this value in the sensitivity analysis.

For the additional scenarios in which we restricted the possibility of women undergoing invasive testing without having NIPT first we assumed that the uptake of NIPT among women with a high risk result (≥1/150) would be 91%. In the RAPID study several very high risk women underwent invasive testing directly, and therefore the proportion of positive NIPT results was expected to be higher when these women were to undergo NIPT first. We estimated an average of 7.9% positive NIPT results for this scenario.

Costs

To assess the costs of NIPT we have taken into account the costs of all different tests (combined/quadruple Down syndrome screening tests, NIPT and invasive testing), including sampling, laboratory testing, and feeding back the results. The unit costs are listed in Table 2 (main paper). Costs of screening were taken from the UK National Screening Committee decision planning tool and inflated to 2012-13 UK£ [15]. Laboratory charges for NIPT (£250) in the RAPID study were used for the costs of NIPT in the base case and these costs were varied over a wide range in the sensitivity analysis. Costs for counselling and feedback by a midwife as well as phlebotomy costs were included [16, 17]. Total charges for invasive testing were collected from the eight participating clinics and the weighted mean unit cost of CVS/QF-PCR and amniocentesis/full karyotype was used; this included pre-test counselling, consultant obstetrician time to perform the procedure, cytogenetic laboratory costs, and post-test feedback and counselling. This value was varied in the sensitivity analysis. All costs are expressed in 2012-13 UK£. The time horizon in this study was the duration of pregnancy, therefore no discounting was necessary.

There were a small number of failed and inconclusive tests in the RAPID evaluation study (n=31 out of 1971 tests performed, excluding the period 26/09/2014 to 10/10/2014 when the study was suspended due to failure of and subsequent unavailability of library preparation kits) Of the women that were offered a repeat test, 79% accepted to have a repeat test. For the purpose of the analysis, we assumed that 1.2% (79% of 31/1971) of NIPT tests would result in a failed/inconclusive test and all these women would undergo a repeat test. To capture this we inflated the costs of NIPT by the proportion of patients requiring extra tests. Because of the small numbers, we did not take into account a difference in costs between rerunning the sample in the lab or redrawing blood and running the test using the new sample.

Model outputs

The main outcomes of the analysis were: screening and diagnosis-related costs of the different pathways; number of Down syndrome cases detected; and, number of miscarriages avoided. Uncertainty around our input parameters was taken into account using one-way sensitivity analysis

and probabilistic sensitivity analysis. In the one-way sensitivity analysis we varied one parameter at a time over a plausible range (mostly equal to 95% confidence limits, but for some parameters such as uptake of screening wider intervals were used) to identify the maximum value at which introducing NIPT to the national screening programme would be cost neutral. In the probabilistic sensitivity analysis, 1000 simulations of the outputs were produced, based on drawing random samples from the probability distributions of all input parameters. Beta distributions were used for most probabilities, Dirichlet distributions for probabilities if there were 3 or more possible outcomes and gamma distributions for all costs. The distribution of the 1000 simulations was used to calculate 95% uncertainty intervals for each of the model outputs.

Type of clinic

We expect a difference in NIPT uptake between one stop clinics (where NIPT is done on the same day as screening) and clinics where women need to come back for NIPT at a later date (screening results are phoned out). In a previous analysis (NSC report) we estimated the uptake of NIPT and direct IPD in both types of clinics. We applied these estimates to our current central estimate as shown in the main paper. As might be expected, in the RAPID study, the uptake of NIPT in women with a risk between 1/150 and 1/1000 was higher in the one-stop clinics than in the clinics where an extra visit for NIPT was required (74.4% versus 59.3%). However, in one stop clinics, high risk women (≥1/150) were more likely to undergo invasive testing directly after screening (23.0% versus 13.9%) and less likely to undergo NIPT first (70.0% versus 77.7%) (Table S1). The reason for this is unclear. Because of this difference, we ran the analysis separately for both types of clinic in a sensitivity analysis. The costs of the different steps in the pathways were assumed to be the same in each type of clinic, because the NIPT test and counselling is charged as a separate contact in both types of clinics. The total costs of the NIPT pathways and the number of cases detected and the number of miscarriages were expected to differ between the two clinics, because of the difference in uptake of NIPT. Because no extra visit for NIPT is required in the one-stop clinic, the costs incurred by the woman are expected to be lower.

References

1. Ayres, A.C., J.A. Whitty, and D.A. Ellwood, *A cost-effectiveness analysis comparing different strategies to implement noninvasive prenatal testing into a Down syndrome screening program.* Aust N Z J Obstet Gynaecol, 2014. **54**(5): p. 412-7.
2. Beulen, L., Grutters, JP., Faas, BH., et al., *The consequences of implementing non-invasive prenatal testing in Dutch national health care: a cost-effectiveness analysis.* Eur J Obstet Gynecol Reprod Biol, 2014. **182c**: p. 53-61.
3. Cuckle, H., P. Benn, and E. Pergament, *Maternal cfDNA screening for Down syndrome--a cost sensitivity analysis.* Prenat Diagn, 2013. **33**(7): p. 636-42.
4. Garfield, S.S. and S.O. Armstrong, *Clinical and cost consequences of incorporating a novel non-invasive prenatal test into the diagnostic pathway for fetal trisomies.* J Managed Care Med. **15**: p. 34-41.
5. Morris, S., Karlsen, S., Chung, N., Hill, M., Chitty, LS. *Model-based analysis of costs and outcomes of non-invasive prenatal testing for Down's syndrome using cell free fetal DNA in the UK National Health Service.* PLoS One, 2014. **9**(4): p. e93559.
6. Neyt, M., F. Hulstaert, and W. Gyselaers, *Introducing the non-invasive prenatal test for trisomy 21 in Belgium: a cost-consequences analysis.* BMJ Open, 2014. **4**(11): p. e005922.
7. Ohno, M. and A. Caughey, *The role of noninvasive prenatal testing as a diagnostic versus a screening tool--a cost-effectiveness analysis.* Prenat Diagn, 2013. **33**(7): p. 630-5.
8. Okun, N., Teitelbaum, M., huang, T., Dewa, CS., Hoch, JS. *The price of performance: a cost and performance analysis of the implementation of cell-free fetal DNA testing for Down syndrome in Ontario, Canada.* Prenat Diagn, 2014. **34**(4): p. 350-6.
9. O'Leary, P., Maxwell, S., Murch, A. *Prenatal screening for Down syndrome in Australia: costs and benefits of current and novel screening strategies.* Aust N Z J Obstet Gynaecol, 2013. **53**(5): p. 425-33.
10. Palomaki, G.E., Kloza, EM., Lambert-Messerlain, GM. *DNA sequencing of maternal plasma to detect Down syndrome: an international clinical validation study.* Genet Med, 2011. **13**(11): p. 913-20.
11. Song, K., T.J. Musci, and A.B. Caughey, *Clinical utility and cost of non-invasive prenatal testing with cfDNA analysis in high-risk women based on a US population.* J Matern Fetal Neonatal Med, 2013. **26**(12): p. 1180-5.
12. Wald, N.J. and J.P. Bestwick, *Incorporating DNA sequencing into current prenatal screening practice for Down's syndrome.* PLoS One, 2013. **8**(3): p. e58732.
13. Walker, B.S., Jackson, BR., LaGrave D. *A cost-effectiveness analysis of cell free DNA as a replacement for serum screening for Down syndrome.* Prenat Diagn, 2014.
14. *NICE National Institute of Health and Care Excellence. Guide to the methods of technology appraisal 2013 (Available from: http://www.nice.org.uk/media/D45/1E/GuideToMethodsTechnologyAppraisal2013.pdf).*
15. *NHS Fetal Anomaly Screening Programme (2012). Decision planning tool. Available from URL:*

http://fetalanomaly.screening.nhs.uk/professionalresources#fileid11456.

16. *Department of Health. National schedule of reference costs 2012/13. Available from URL:*

https://www.gov.uk/government/publications/nhs-reference-costs-2012-to-2013.

17. Curtis, L., *Unit Costs of Health and Social Care 2013.* Personal Social Services Research Unit; University of Kent, 2013.
18. *Down's Syndrome Screening Quality Assurance Support Service (DQASS).*

19. *The National Down Syndrome Cytogenetic Register for England and Wales: 2013 Annual Report.*
20. Henderson, J., Bricker L., Roberts, T. *British National Health Service's and women's costs of antenatal ultrasound screening and follow-up tests.* Ultrasound Obstet Gynecol, 2002. **20**(2): p. 154-62.
21. *Office for National Statistics. Annual survey of Hours and Earnings, 2013 Provisional Results.*
22. *AA Motoring costs 2013. Available from URL: https://www.theaa.com/resources/Documents/pdf/motoring-advice/running-costs/petrol2013.pdf.*
23. Propper, C., Damiani, M., Leckie, G. *Distance Travelled in the NHS in England for Inpatient Treatment.* CMPO Working Paper Series No. 06/162, 2006.

Table S1. Uptake parameters for one stop clinics and two stop clinics

Parameter	One stop	Two stop
**Uptake**		
NIPT after ≥1/150 risk	70.0%	77.7%
NIPT after 1/151- 1/1000 risk	74.4%	59.3%
NIPT after ≥1/150 risk - No direct IPD	90.9%	91.3%
Directly to IPD after ≥1/150 risk	23.0%	13.9%

Table S2. Costs and outcomes of each pathway (in a screening population of 698,500 pregnant women) - using RAPID data

	Screening			NIPT (£250)		Invasive testing			Total costs	DS positive		IPD related miscarriage
Testing strategy	No	Test +ive	£000	No	£000	direct No	after NIPT No	£000	£000	NIPT or IPD	Confirmed by IPD	No
Current	549835	14846	£15,748	0	£0	8017	0	£5,211	£20,959	806	806	40
NIPT ≥1/1000	549835	53884	£15,748	38285	£10,643	2969	402	£2,191	£28,582	1146	1010	17
NIPT ≥1/500	549835	32440	£15,748	23167	£6,440	2969	384	£2,180	£24,368	1125	995	17
NIPT ≥1/150	549835	14846	£15,748	10763	£2,992	2969	346	£2,155	£20,895	1078	960	17
NIPT ≥1/1000 - No direct IPD	549835	53884	£15,748	41031	£11,406	0	914	£594	£27,749	1136	827	5
NIPT ≥1/500 - No direct IPD	549835	32440	£15,748	25914	£7,204	0	896	£583	£23,535	1114	811	4
NIPT ≥1/150 - No direct IPD	549835	14846	£15,748	13509	£3,756	0	858	£558	£20,062	1067	776	4

Table S3. Incremental costs and outcomes compared to the current pathway (in a screening population of 698,500 pregnant women) - using RAPID data

	Per 698,500 pregnant women (95% uncertainty interval [lower limit ; upper limit])				
Testing strategy	Incremental DS positive NIPT or IPD	Incremental DS confirmed by IPD	IPD avoided	IPD related miscarriage avoided	Incremental costs [at NIPT test costs £250] (£000)
NIPT ≥1/1000	341 (-30 ; 718)	205 (-153 ; 556)	4646 (3404 ; 5874)	23.2 (10.2 ; 41.7)	£7623 (£857 ; £18287)
NIPT ≥1/500	319 (-61 ; 704)	189 (-160 ; 539)	4663 (3425 ; 5870)	23.3 (10.5 ; 42.7)	£3409 (£-1013 ; £9851)
NIPT ≥1/150	272 (-73 ; 612)	155 (-168 ; 460)	4701 (3886 ; 5544)	23.5 (10.6 ; 41.3)	£-64 (£-2579 ; £3131)
NIPT ≥1/1000 - No direct IPD	330 (-12 ; 706)	21 (-341 ; 355)	7103 (6064 ; 8175)	35.5 (16.0 ; 62.5)	£6790 (£-741 ; £18008)
NIPT ≥1/500 - No direct IPD	309 (-51 ; 673)	5 (-351 ; 345)	7120 (6067 ; 8198)	35.6 (16.1 ; 62.7)	£2576 (£-2662 ; £10066)
NIPT ≥1/150 - No direct IPD	262 (-81 ; 608)	-29 (-363 ; 264)	7158 (6212 ; 8141)	35.8 (16.1 ; 62.9)	£-897 (£-4401 ; £3292)

Table S4. One-way sensitivity analysis. This table shows threshold values at which the different NIPT pathways would be cost neutral compared with the current pathway - Using RAPID data

Parameter	Range for one-way SA	Threshold values 1/1000	1/500	1/150	1/1000 - no direct IPD	1/500 - no direct IPD	1/150 - no direct IPD
**Screening test performed**							
Combined test (first trimester)	80-95%	*	*	-	*	*	-
**Uptake**							
DSS - current pathway	50-100%	*	92%	78%	*	88%	75%
DSS - NIPT pathway	50-100%	58%	68%	79%	59%	70%	82%
NIPT after ≥1/150 risk	70-85%	*	*	71%	*	*	-
NIPT after 1/151- 1/1000 risk	75-90%	*	*	-	*	*	-
NIPT after ≥1/150 risk - No direct IPD	80-100%	*	*	-	*	*	-
IPD after positive screening (current)	50-65%	*	*	53%	*	*	-
Directly to IPD after ≥1/150 risk (NIPT)	10-25%	*	*	21%	*	*	-
IPD after positive NIPT	70-100%	*	*	-	*	*	-
**Test outcomes**							
Women with DSS risk ≥1/150	2-3.5%	*	*	-	*	*	-
Women with DSS risk 1/151- 1/500	3-5%	*	*	-	*	*	-
Women with DSS risk 1/151- 1/1000	7-10%	*	*	-	*	*	-
NIPT positive when DSS ≥1/150 and accepted NIPT	3-9%	*	*	5.1%	*	*	-
NIPT positive when DSS 1/151- 1/500 and accepted NIPT	0.1-1%	*	*	-	*	*	-
NIPT positive when DSS 1/151- 1/1000 and accepted NIPT	0.1-1%	*	*	-	*	*	-
NIPT positive when DSS ≥1/150 and accepted NIPT - No direct IPD	6-14%	*	*	-	*	*	-
NIPT failure followed by repeat test	1-3%	*	*	-	*	*	-
IPD positive if accepted IPD - current pathway	4-20%	*	*	-	*	*	-
IPD positive if accepted IPD after positive NIPT	80-100%	*	*	-	*	*	-
IPD positive - directly to IPD ≥1/150	15-40%	*	*	-	*	*	-
IPD related miscarriage	0.3-1%	*	*	-	*	*	-
**Costs**							
Combined test	£15-£40	*	*	-	*	*	-
Quadruple test	£20-£55	*	*	-	*	*	-
Laboratory costs NIPT	£50-£500	£53	£105	£256	£87	£152	£316
Costs midwife for counselling and feedback	£10-£25	*	*	£22	*	*	-
Costs phlebotomy and sending in sample	£5-£15	*	*	£15	*	*	-
Cost of invasive test	370-1000	*	*	£636	*	*	£525

- means that varying the parameter within the specified range did not change the results from cost saving to cost neutral, * means that varying the parameter within the specified range did not change the results from increased costs to cost neutral

Table S5. One-way sensitivity analysis. This table shows threshold values at which the different NIPT pathways would be cost neutral compared with the current pathway- Using national data

Parameter	Range for one-way SA	Threshold values 1/1000	1/500	1/150	1/1000 - no direct IPD	1/500 - no direct IPD	1/150 - no direct IPD
**Screening test performed**							
Combined test (first trimester)	80-95%	*	*	-	*	*	-
**Uptake**							
DSS - current pathway	50-100%	93%	78%	66%	90%	76%	64%
DSS - NIPT pathway	50-100%	*	56%	66%	*	58%	69%
NIPT after ≥1/150 risk	70-85%	*	*	71%	*	*	-
NIPT after 1/151- 1/1000 risk	75-90%	*	*	-	*	*	-
NIPT after ≥1/150 risk - No direct IPD	80-100%	*	*	-	*	*	-
IPD after positive screening (current)	50-65%	*	*	53%	*	*	-
Directly to IPD after ≥1/150 risk (NIPT)	10-25%	*	*	21%	*	*	-
IPD after positive NIPT	70-100%	*	*	-	*	*	-
**Test outcomes**			*	-	*	*	-
Women with DSS risk ≥1/150	2-3.5%	*	*	-	*	*	-
Women with DSS risk 1/151- 1/500	3-5%	*	*	-	*	*	-
Women with DSS risk 1/151- 1/1000	7-10%	*	*	-	*	*	-
NIPT positive when DSS ≥1/150 and accepted NIPT	3-9%	*	*	5.1%	*	*	-
NIPT positive when DSS 1/151- 1/500 and accepted NIPT	0.1-1%	*	*	-	*	*	-
NIPT positive when DSS 1/151- 1/1000 and accepted NIPT	0.1-1%	*	*	-	*	*	-
NIPT positive when DSS ≥1/150 and accepted NIPT - No direct IPD	6-14%	*	*	-	*	*	-
NIPT failure followed by repeat test	1-3%	*	*	-	*	*	-
IPD positive if accepted IPD - current pathway	4-20%	*	*	-	*	*	-
IPD positive if accepted IPD after positive NIPT	80-100%	*	*	-	*	*	-
IPD positive - directly to IPD ≥1/150	15-40%	*	*	-	*	*	-
IPD related miscarriage	0.3-1%	*	*	-	*	*	-
**Costs**							
Combined test	£15-£40	*	*	-	*	*	-
Quadruple test	£20-£55	*	*	-	*	*	-
Laboratory costs NIPT	£50-£500	£42	£89	£256	£71	£133	£316
Costs midwife for counselling and feedback	£10-£25	*	*	£22	*	*	-
Costs phlebotomy and sending in sample	£5-£15	*	*	£15	*	*	-
Cost of invasive test	370-1000	*	*	£636	*	*	£525

- means that varying the parameter within the specified range did not change the results from cost saving to cost neutral, * means that varying the parameter within the specified range did not change the results from increased costs to cost neutral

Table S6. Incremental costs compared to the current pathway (in a screening population of 698,500 pregnant women) for a range of uptake values for NIPT and IPD in high-risk pregnancies - using RAPID data

	Uptake			Incremental costs [at NIPT test cost £250] (£000)	
**Scenario**	**NIPT**	**IPD**	**Total**	**1/150**	**1/150 - no direct IPD**
Main analysis	72.5%	20.0%	91.0%	-£64	-£897
Lowest NIPT uptake (WHIT)	68.5%	25.9%	94.4%	£328	-£736
Highest NIPT uptake & highest total uptake (SAL)	96.6%	3.4%	100.0%	-£596	-£471
Lowest total uptake (QHR)	74.5%	13.9%	88.5%	-£564	-£1,016

Uptake figures were derived from Table 1 in the main text but including T21 only.

Figure S1. Benefits and costs of the DS screening pathway nationally for the current pathway and using NIPT as a contingent test for women with a DSS risk of ≥ 1/150, 1/500 and 1/1000.

RAPID DATA

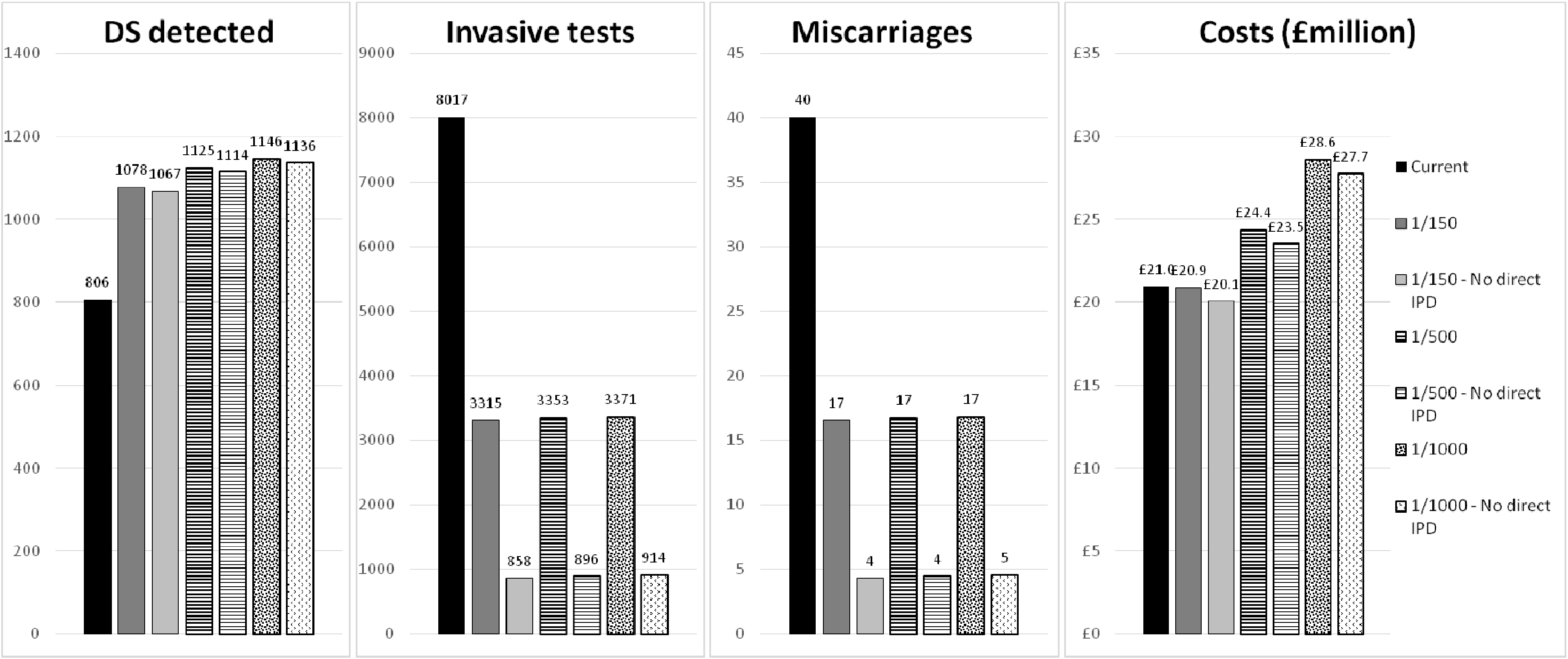

Figure S2. Marginal costs for different base prices of implementing NIPT as a contingent test for DS for women with a DS screening risk $\geq$ 1 in 150 (▲), 1 in 500 (■), or 1 in 1000 (●). The dashed line indicates costs without the option of direct IPD and the other lines costs allowing the option of direct IPD without prior NIPT.

RAPID DATA

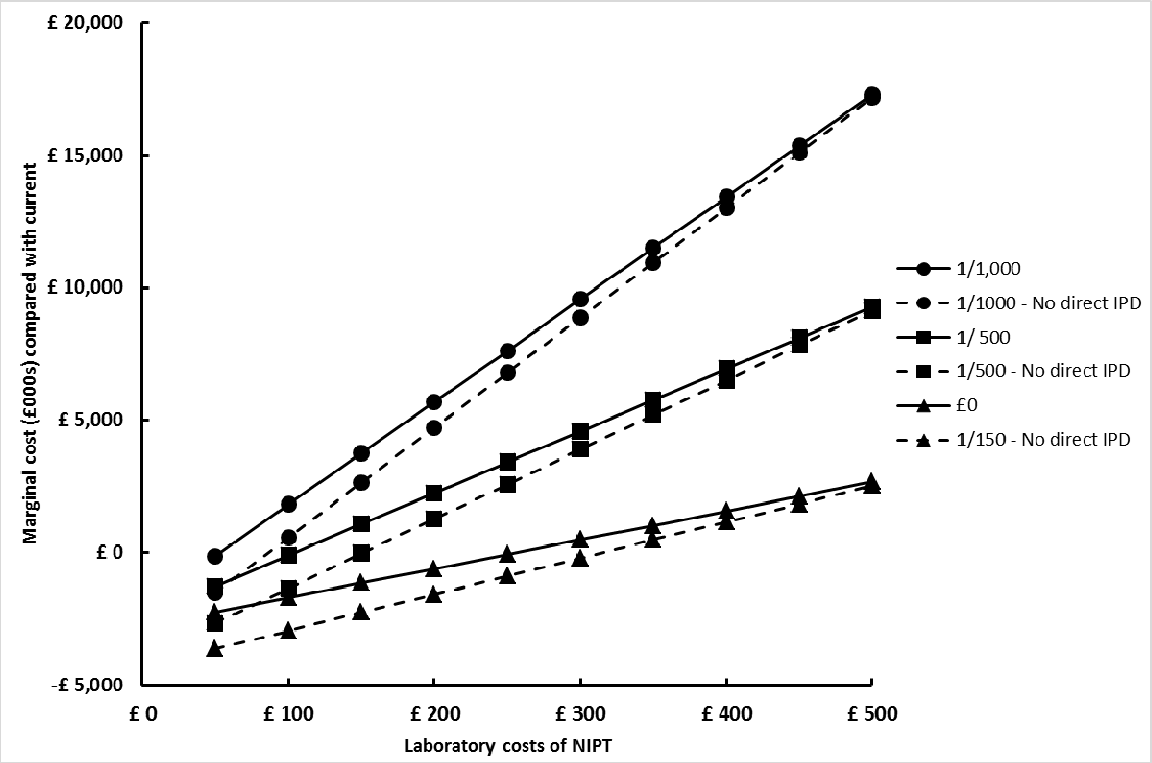

NATIONAL DATA

Figure S3a. Benefits and costs of the DS screening pathway nationally for the current pathway and using NIPT as a contingent test for women with a DSS risk of ≥ 1/150, 1/500 and 1/1000. **One stop clinics**

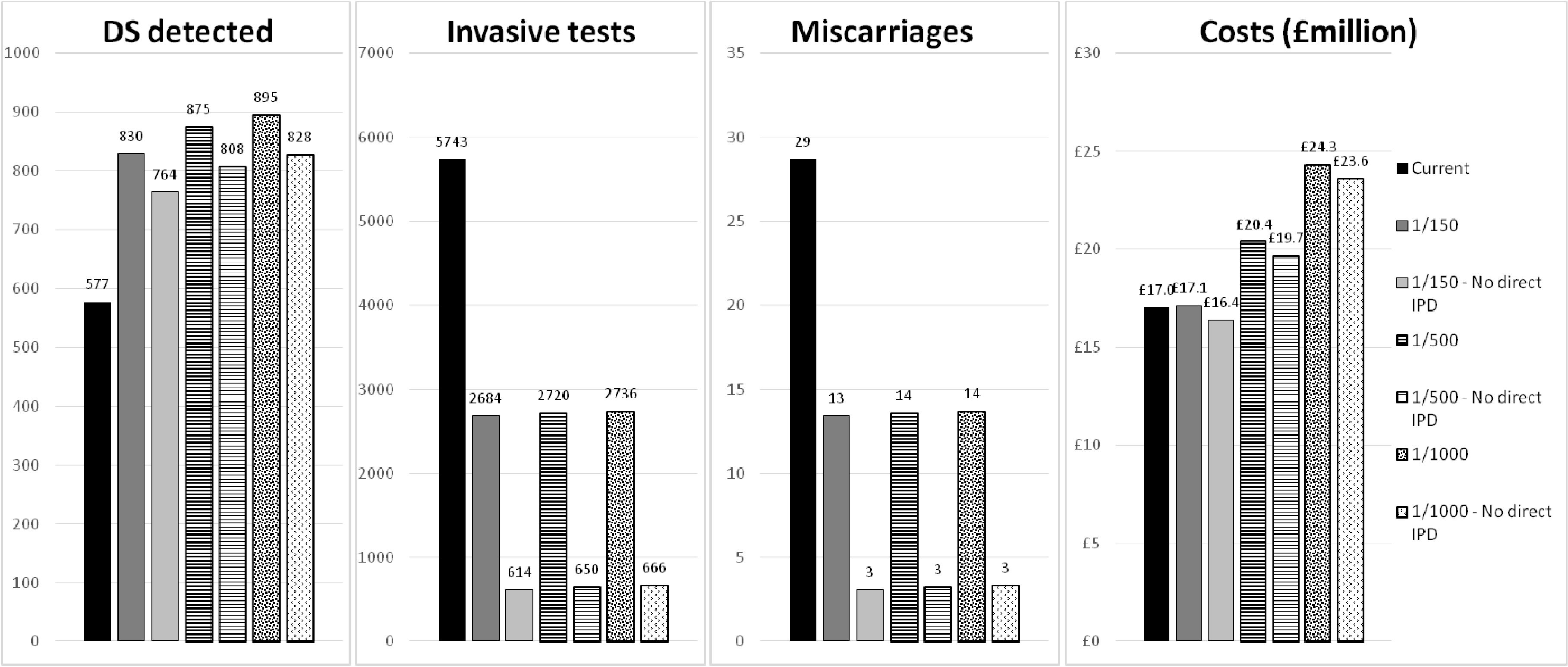

Figure S3b. Benefits and costs of the DS screening pathway nationally for the current pathway and using NIPT as a contingent test for women with a DSS risk of ≥ 1/150, 1/500 and 1/1000. **Two stop clinics**

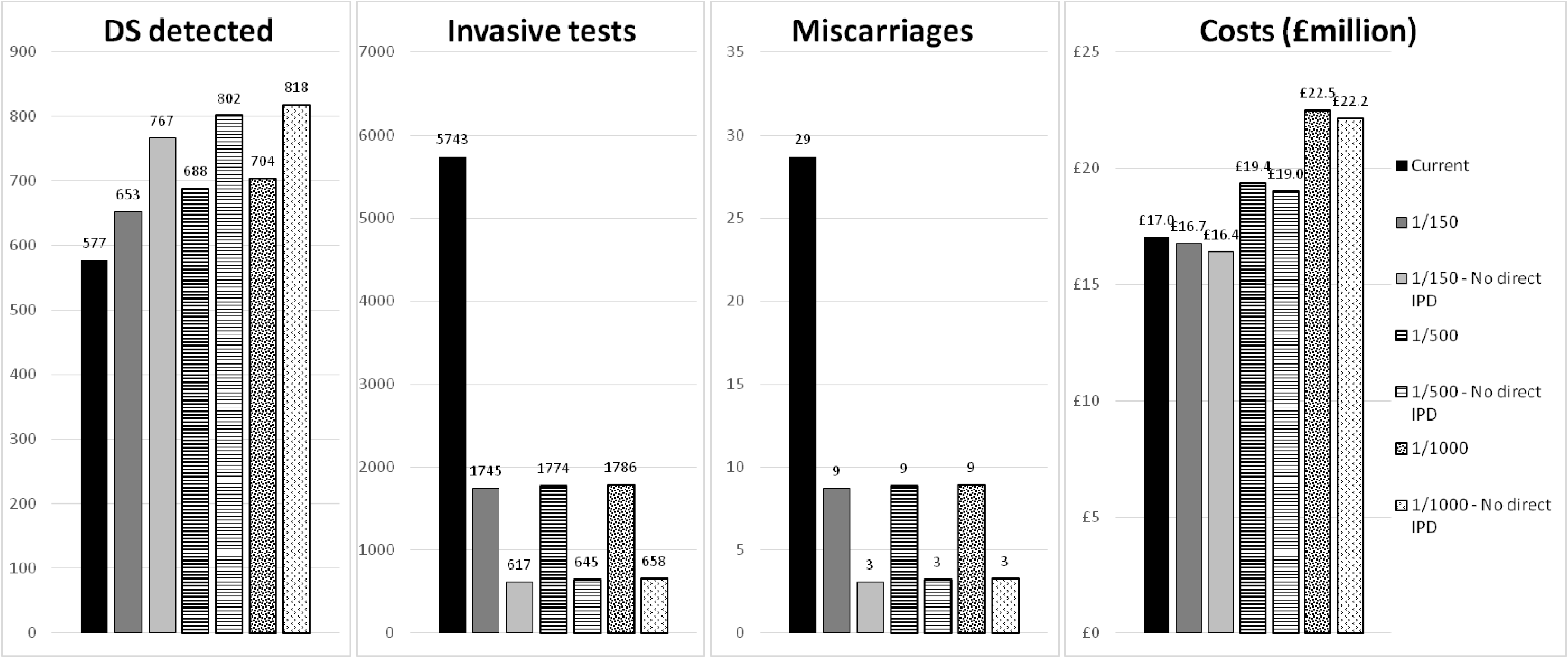

RAPID DATA

Figure S4a. Benefits and costs of the DS screening pathway nationally for the current pathway and using NIPT as a contingent test for women with a DSS risk of > 1/150, 1/500 and 1/1000. **One stop clinics**

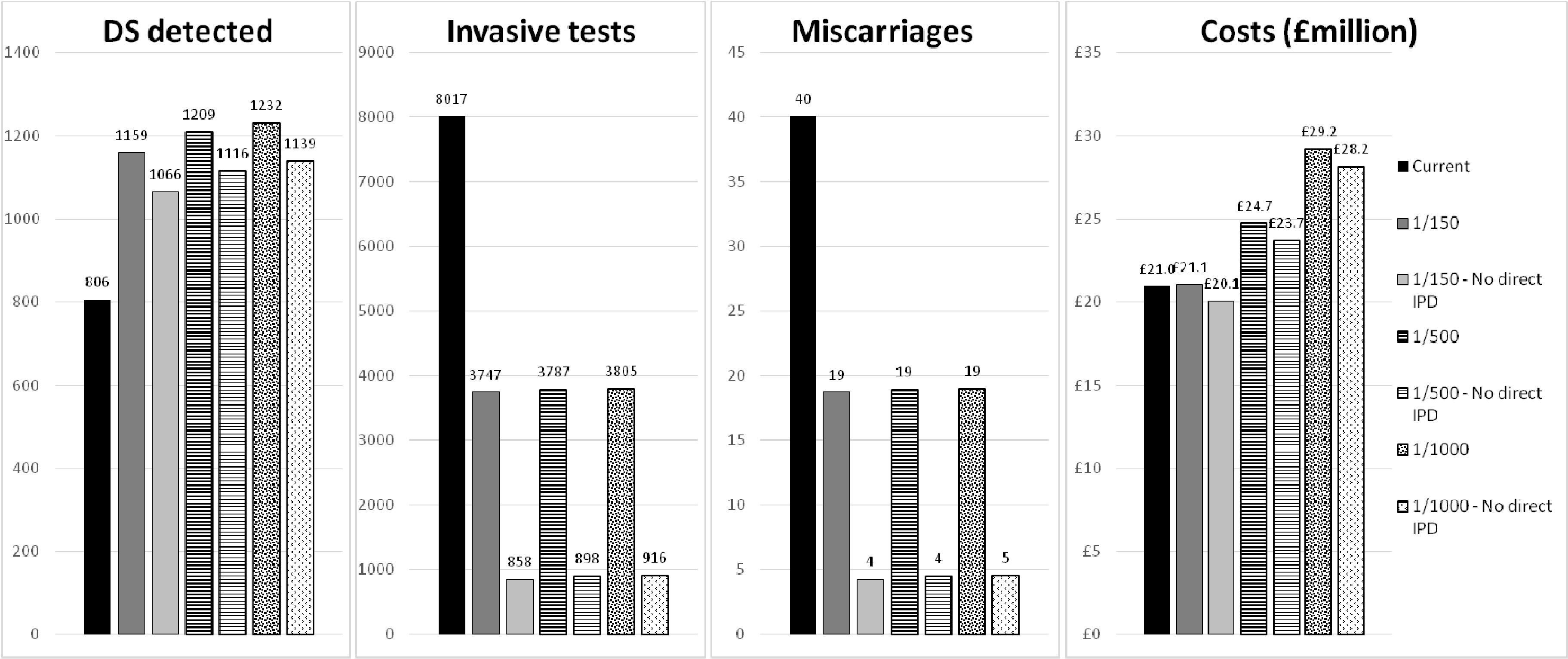

Figure S4b. Benefits and costs of the DS screening pathway nationally for the current pathway and using NIPT as a contingent test for women with a DSS risk of ≥ 1/150, 1/500 and 1/1000. **Two stop clinics**

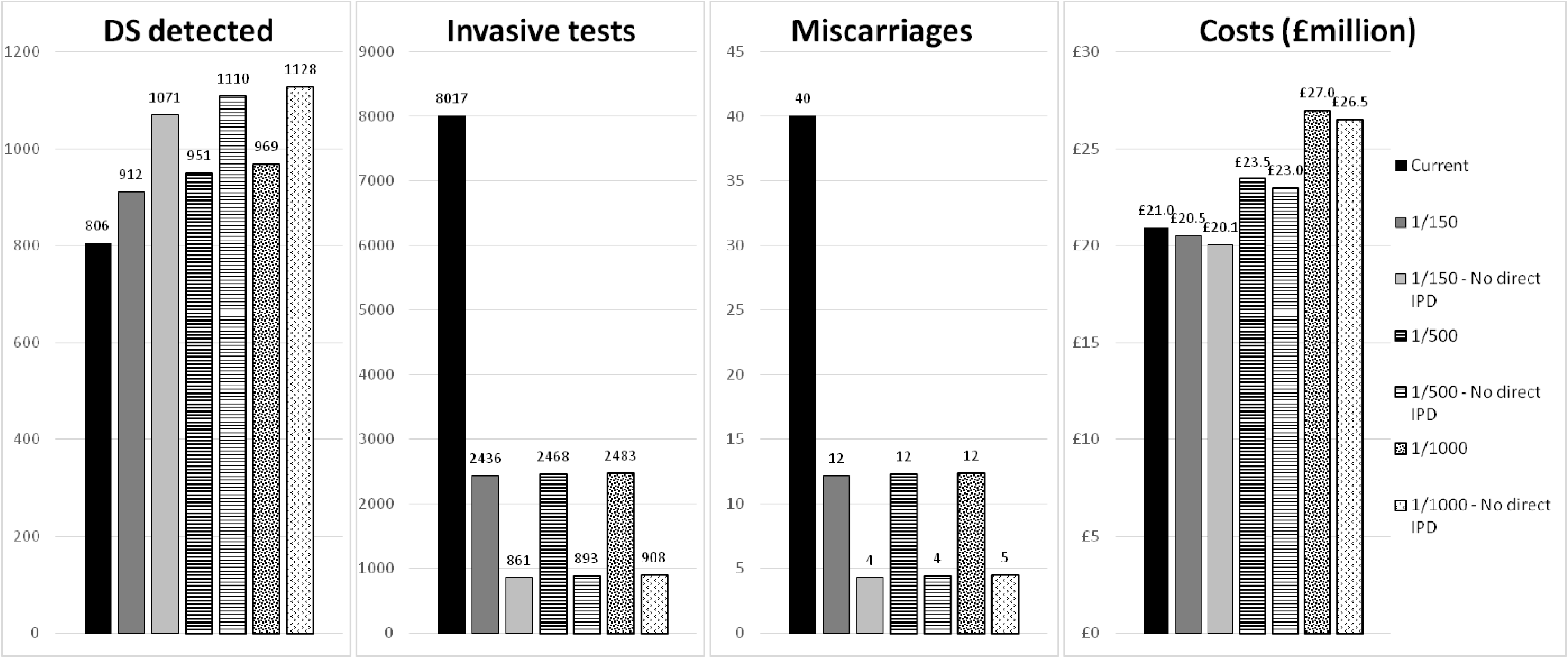

Supplement: Supplementary file 3 — Appendix C [file chil030961.ww3_default.pdf]
