# Supplementary material for: Uptake, outcomes, and costs of implementing non-invasive prenatal testing for Down’s syndrome into NHS maternity care: prospective cohort study in eight diverse maternity units
Source: BMJ. 2016 Jul 4;354:i3426. doi: 10.1136/bmj.i3426 (PMC4933930; doi:10.1136/bmj.i3426)
Supplement: Supplementary file 4 — Appendix D [file chil030961.ww4_default.pdf]

Modelling Group Probabilities

The health economic model is populated in terms of the probabilities of DS given risk group and acceptance of further testing by NIPT or by IPD as appropriate. For women in a specific risk group (e.g. DS risk of 1 in 150 or higher) who accept NIPT, the model requires the conditional probability of DS. When comparing different policies, it is important that the probabilities reflect the same underlying population prevalence appropriate for the purposes of the health economic assessment. To achieve this, we assumed the maternal age distribution of England and Wales 2011 denoted by $p(m)$ and assumed that that the prevalence of DS arose according to the model of Wright and Bray, 2000. In this model the term risk of Downs syndrome $p(DS|m)$ is an increasing function of maternal age m.

The probabilities required for the health economic model are, for example $p\big(\text{Accept NIPT}|R \geq \frac{1}{150}\big)$ and $p\big(DS|R \geq \frac{1}{150} \text{ and accept NIPT}\big)$ where R denotes the combined test risk. It is possible to estimate $p\big(\text{Accept NIPT}|R \geq \frac{1}{150}\big)$ using the sample proportion of women in the high risk group who choose NIPT. However, estimation of $p\big(DS|R \geq \frac{1}{150} \text{ and accept NIPT}\big)$ is problematic because of selection bias and the relatively small number of cases in the data set especially in the lower risk groups. The RAPID date were used to model the

probabilities of direct choice of IPD for those with risk of $\frac{1}{150}$ or higher and the choice of NIPT in for those with risks of $\frac{1}{1{,}000}$ or higher. This was achieved using a logistic regression on the logit transformation of risk. Results for acceptance of NIPT and for acceptance of IPD are given in Tables 1 and 2 respectively. The fitted probabilities are shown in Figure 1. As expected, acceptance of further testing increases with risk and acceptance of NIPT is higher than acceptance IPD.

	Estimate	Std. Error	z value	Pr(>|z|)
Intercept	3.498333	0.275218	12.711123	<0.000001
logit(DS risk)	0.322656	0.046512	6.936969	<0.000001

Table 1: Logistic regression model for acceptance of NIPT following combined testing

	Estimate	Std. Error	z value	Pr(>|z|)
Intercept	1.325799	0.399697	3.317013	0.000910
logit(DS Risk)	0.928137	0.093364	9.941106	<0.000001

Table 2: Logistic regression model for acceptance of IPD following combined testing

In order to populate the health economic model in such a way that different policies were assessed against the same reference population, we used the probability calculations below.

Acceptance if NIPT given risk group

$$p(\text{Accept NIPT}|G) = \int_G p(\text{Accept NIPT}|R)p(R)dR \tag{1}$$

where G denotes an interval for the risk R, e.g. $0 < G < 1/150$.

DS given risk group and acceptance of NIPT

$$p(DS|\text{Accept NIPT and } G) = \frac{\int_G p(DS|R)p(\text{Accept NIPT}|R)p(R)dR}{p(\text{Accept NIPT}|G)} \tag{2}$$

where G denotes an interval for the risk R, e.g. $0 < G < 1/150$.

The distribution of risk R was obtained from combined test distributional parameters with the maternal age distribution of England and Wales 2011. Maternal specific prevalence of DS was obtained from Wright and Bray 2000.

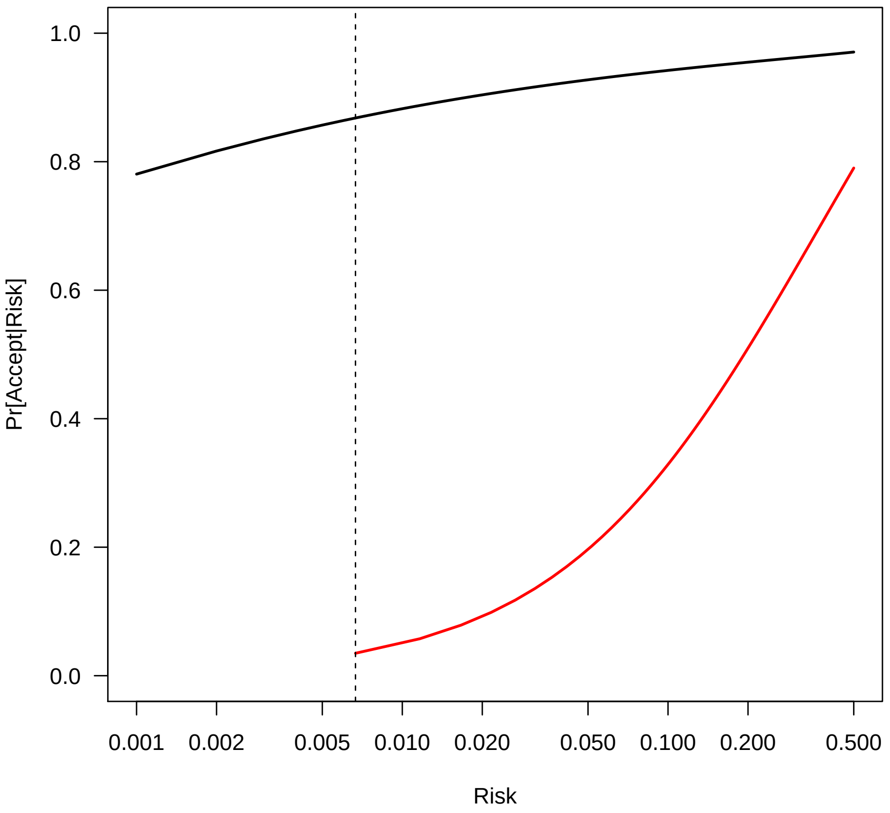

Figure 1: Acceptance probabilities following combined testing by DS risk for NIPT (black) and IPD (red).

Reference

Wright, D. and I. Bray. Estimating birth prevalence of Downs syndrome. Journal of Epidemiology and Biostatistics 2000; 5: 89-98.
